# Multimodal Canonical Correlation Analysis with Joint Independent Component Analysis (mCCA+jICA) of IVIM and ASL MRI Reveals Perfusion and Diffusion Abnormalities in mTBI—A Pilot Study

**DOI:** 10.3390/neurosci6040123

**Published:** 2025-12-03

**Authors:** Maurizio Bergamino, Lauren R. Ott, Molly M. McElvogue, Ruchira Jha, Cindy Moreno, Ashley M. Stokes

**Affiliations:** Barrow Neuroimaging Innovation Center, Barrow Neurological Institute, Phoenix, AZ 85013, USA; lauren.ott@commonspirit.org (L.R.O.); molly.mcelvogue@commonspirit.org (M.M.M.); ruchira.jha@barrowneuro.org (R.J.); cindy.moreno@commonspirit.org (C.M.); ashley.stokes@barrowneuro.org (A.M.S.)

**Keywords:** multimodal canonical correlation analysis, joint independent component analysis, intravoxel incoherent motion, arterial spin labeling, cerebral blood flow, concussion, mTBI

## Abstract

Mild traumatic brain injury (mTBI) frequently causes subtle brain changes that are difficult to detect with conventional diagnostic approaches. In this exploratory pilot study, we combined tri-exponential intravoxel incoherent motion (IVIM) and pseudocontinuous arterial spin labeling (pCASL) MRI with Multimodal Canonical Correlation Analysis and joint independent component analysis (mCCA+jICA) to identify imaging signatures distinguishing mTBI patients from healthy controls (HCs) and their associations with clinical function. Cerebral blood flow (CBF) and IVIM-derived metrics were extracted from 90 brain regions in 19 mTBI patients and 24 HCs, and multivariate components were identified using mCCA+jICA. Two independent components (IC2, IC15) showed group differences at the uncorrected level (*p* < 0.05) but did not survive false discovery rate (FDR) correction. IC2 correlated positively with CBF and perfusion fraction (*F_p_*) and negatively with tissue diffusion fraction (*F_s_*), consistent with reduced vascular integrity in mTBI, while IC15 showed similar trends. One component correlated with Glasgow Outcome Scale–Extended (GOS-E) scores (uncorrected *p* = 0.046). Although this study is preliminary and limited by a small sample size, our findings suggest that mTBI is associated with perfusion and microstructural alterations, particularly in subcortical regions, and demonstrate the potential value of combining IVIM and ASL within multivariate fusion frameworks to reveal patterns not captured by single-modality approaches.

## 1. Introduction

Mild traumatic brain injury (mTBI) encompasses a range of injuries caused by biomechanical forces impacting the head, often leading to a disruption in normal brain function [1]. mTBI accounts for over 70% of all traumatic brain injuries [2], with most cases resulting from falls, motor vehicle accidents, or sports-related impacts. Concussion represents the most common form of mTBI and is typically associated with transient neurological symptoms such as headache, dizziness, and cognitive disturbances. However, mTBI as a whole encompasses a broader spectrum of clinical presentations, and in some cases, mTBI may lead to persistent symptoms and subtle alterations in brain structure and function that are often difficult to detect with conventional neuroimaging [1,3]. Advanced imaging techniques that can detect subtle brain changes associated with mTBI are of increasing interest for their potential to enhance diagnostic accuracy, improve prognostic modeling, and inform individualized treatment strategies.

Among advanced imaging modalities, magnetic resonance imaging (MRI) has become a cornerstone for assessing the structural and functional consequences of mTBI. Diffusion MRI (dMRI), for instance, has revealed alterations in white matter integrity in mTBI patients [4], with diffusion tensor imaging (DTI) metrics serving as potential indicators of axonal injury or demyelination [5]. Perfusion-based MRI approaches have also detected hemodynamic deficits in concussion, highlighting possible cerebrovascular dysregulation [6]. Increasingly, multimodal neuroimaging is employed to capture complementary dimensions of mTBI pathophysiology. While diffusion metrics are sensitive to microstructural changes, perfusion imaging provides information on cerebrovascular health and metabolic demand. Integrating these approaches has been shown to reveal abnormalities that single modalities may miss, such as concurrent white matter microstructural disruption and regional hypoperfusion [7,8,9].

While these imaging biomarkers have provided insight into the pathophysiology of concussion and mTBI, they also present key limitations. In particular, the interpretation of DTI metrics is constrained by the assumption of Gaussian diffusion and the challenge of disentangling signal contributions from complex tissue environments and perfusion effects [10]. For this reason, there is growing interest in advanced diffusion models that can more accurately capture the heterogeneity of brain microstructure.

The intravoxel incoherent motion (IVIM) dMRI model incorporates both molecular diffusion and microvascular perfusion into the diffusion-weighted signal model to improve the biological interpretability of dMRI [11]. The traditional bi-exponential IVIM model is an MRI technique that separates the overall diffusion signal into two distinct components: a rapid component, known as pseudo-diffusion, which primarily reflects the random microscopic motion of blood within capillaries (i.e., perfusion), and a slower component corresponding to true tissue diffusion, representing the hindered motion of water molecules within the tissue cellular and extracellular spaces [12]. Recent studies applying the bi-exponential IVIM model have demonstrated complementary diffusion and perfusion changes in mild cognitive impairment (MCI) and Alzheimer’s disease (AD), both cross-sectionally [13,14] and longitudinally [15], as well as in brain tumors [16].

To improve compartmental specificity and better reflect free-water and restricted diffusion environments, a tri-exponential IVIM model was previously introduced to further differentiate between multiple compartments within tissue [17,18]. This advanced approach typically decomposes the diffusion signal decay into three compartments, each represented by a diffusion coefficient and associated volume fraction: a slow diffusion component (*D_s_* and *F_s_*) reflecting true tissue diffusion; an intermediate diffusion compartment (*D_f_* and *F_f_*) potentially associated with free-water dynamics or pseudo-diffusion; and a fast, perfusion-related compartment (*D_p_* and *F_p_*), typically arising from microvascular blood flow (e.g., capillaries).

To date, the tri-exponential IVIM model has been applied in the brain in only few studies, including healthy controls (HCs) [18,19] and a preliminary study of concussion [20]. The refined compartmentalization from this model improves the characterization of tissue microstructure and vascular dynamics, offering a more nuanced understanding of complex pathological processes such as injury, inflammation, and vascular dysfunction.

In addition to diffusion-based metrics, cerebral perfusion represents a complementary biomarker of interest in mTBI. Arterial spin labeling (ASL) is a non-invasive method that quantifies cerebral blood flow (CBF) by magnetically labeling inflowing arterial blood water as an endogenous tracer [21]. Among various ASL methods, pseudocontinuous ASL (pCASL) has become the most widely used due to its favorable signal-to-noise ratio, labeling efficiency, and compatibility with clinical scanners [22]. pCASL provides quantitative CBF maps without the need for exogenous contrast agents, making it particularly suitable for assessing perfusion abnormalities in conditions like mTBI.

ASL has been applied in both acute and chronic stages of mTBI to investigate changes in regional cerebral perfusion, which may reflect disrupted neurovascular coupling, microvascular injury, or altered metabolic demand following injury. Studies have consistently reported perfusion abnormalities in regions commonly implicated in the pathophysiology of concussion, including regional hypoperfusion in the frontal and temporal lobes, thalamus, and posterior cingulate cortex [23,24,25]. These findings suggest that ASL may serve as a sensitive imaging tool for detecting physiological disruptions in mTBI that are not evident on structural imaging.

In this pilot study, we investigated the differences between healthy individuals and patients with mTBI by combining biomarkers from the tri-exponential IVIM model and pCASL perfusion imaging. Our primary goal was to evaluate the feasibility of combining tri-exponential IVIM and pCASL in mTBI and to identify preliminary trends that will lay the groundwork for larger, confirmatory studies. To analyze these complementary imaging modalities, we employed Multimodal Canonical Correlation Analysis with Joint Independent Component Analysis (mCCA+jICA) [26,27], a data-driven approach that identifies shared and distinct patterns of variance across multimodal datasets. mCCA+jICA outperforms simpler multivariate methods in mTBI by jointly identifying linked patterns across modalities, capturing subtle and spatially heterogeneous brain alterations that single-modality or linear approaches might miss. By integrating IVIM-derived microstructural and perfusion-related parameters with CBF maps from pCASL, this method enables the extraction of “multimodal signatures” that distinguish individuals with mTBI from HCs. Furthermore, we examined the relationship between these imaging-derived components and clinical outcomes, as measured by the Glasgow Outcome Scale–Extended (GOS-E) [28], aiming to uncover latent associations that may be overlooked by unimodal analyses. This integrative approach has the potential to enhance our understanding of the complex pathophysiology of mTBI and its clinical manifestations. Additionally, given the complexity and heterogeneity of mTBI-related brain changes, IVIM imaging, particularly through tri-exponential modeling, presents a novel and potentially more sensitive method for investigating microvascular and microstructural alterations.

## 2. Materials and Methods

### 2.1. Subjects

This study included 24 healthy controls (HCs; 12 females, mean age 29.4 ± 6.2 years) and 19 individuals with mild traumatic brain injury (mTBI; 10 females, mean age 28.0 ± 7.7 years). Within the mTBI group, the primary mechanisms of injury were motor vehicle accidents (*n* = 10) and falls (*n* = 9). Of the 19 mTBI participants, 17 were diagnosed with concussion, and 2 presented with intracranial hemorrhage (one subdural, one subarachnoid). These subgroup counts sum to the total mTBI sample (*n* = 19). All participants with mTBI were recruited from the emergency department, where they were evaluated and diagnosed by a physician based on clinical presentation and standard diagnostic workup prior to recruitment. The mTBI group was assessed using the GOS-E [28], a structured interview that assesses functional recovery after brain injury by categorizing outcomes into eight levels, ranging from death to full recovery, to provide a detailed view of a person’s independence and ability to resume daily activities. Table 1 shows complete demographic and clinical information for both groups.

For the assessment of test–retest reliability of the IVIM/ASL metrics, a subgroup of 11 HCs (4 females, mean age 31.8 ± 6.6 years) underwent two visits separated by 14.1 ± 2.3 days.

### 2.2. MRI Acquisition

MRI data were acquired using a 3.0T Philips Ingenia scanner. Whole-brain T1-weighted Magnetization-Prepared Rapid Gradient Echo (MPRAGE) images were acquired with the following acquisition parameters: field of view (FOV) = 256 × 256 mm^2^, matrix size = 256 × 256, voxel size = 1.0 × 1.0 × 1.0 mm^3^, repetition time (TR) = 7.0 ms, echo time (TE) = 3.2 ms, and flip angle = 9°. Scan time was approximately 6 min and 18 s.

dMRI data were acquired using a modified IVIM protocol consisting of 14 b-values ranging from 10 to 3000 s/mm^2^ and a total of 81 diffusion-weighted directions, plus 2 b0 images. The number of directions per b-value was as follows: 3 directions for b = 10, 25, 35, 50, 75, 100, and 200 s/mm^2^; 10 directions for b = 500 and 750 s/mm^2^; 20 directions for b = 1000 s/mm^2^; and 5 directions for b = 1500, 2000, 2500, and 3000 s/mm^2^. Data were acquired using 2D Echo-Planar Imaging (EPI) with the following acquisition parameters: FOV = 256 × 256 mm^2^, matrix size = 128 × 128, voxel size = 2.0 × 2.0 × 2.0 mm^3^, TR = 7000 ms, TE = 121 ms, and flip angle = 90°. Scan time was approximately 10 min and 3 s. A single reverse-phase dMRI volume with the same acquisition parameters was also acquired to correct EPI distortions (scan time: 42 s).

For perfusion imaging, pCASL was performed based on the recommendations from the ASL consensus paper [21]. Labeling was performed using a parallel slab positioned 90 mm below the imaging volume in the feet-head direction, with imaging slices acquired in the transverse plane. Images were acquired using a 3D gradient and spin echo (GraSE) sequence with the following acquisition parameters: FOV = 224 × 224 mm^2^, acquisition matrix size = 64 × 60, acquisition voxel size = 3.50 × 3.75 × 3.50 mm3, final voxel size = 3.50 × 3.50 × 3.50 mm^3^, TR = 4126 ms, TE = 12 ms, flip angle = 90°, labeling duration = 1800 ms, and post-labeling delay (PLD) = 2000 ms. Scan time was approximately 8 min and 40 s.

### 2.3. Pseudocontinuous ASL (pCASL)

After converting the pCASL and MPRAGE DICOM images to NIfTI format using dcm2niix (https://github.com/rordenlab/dcm2niix (accessed on 2 March 2024)) [29], the pCASL data were corrected for head motion using MCFLIRT (FSL) [30]. The time series were then split into label and control images, and CBF maps were computed in each subject’s native space following the standard single-compartment kinetic model [21] using an in-house bash script.

To enable the transformation of Automated Anatomical Labelling atlas 3 (ALL90, the AAL3 w/o cerebellum areas; https://www.gin.cnrs.fr/en/tools/aal/ (accessed on 15 February 2025)) [31] from MNI space to each subject’s native space, the MPRAGE images were first brain extracted using antsBrainExtraction.sh script in Advanced Normalization Tools (ANTs; https://github.com/ANTsX/ANTs (accessed on 15 February 2025)), then normalized to MNI space using FLIRT (FSL) with 12 degrees of freedom (dof) and trilinear interpolation [32].

Subsequently, a baseline pCASL image was created for each subject by averaging all volumes of the motion-corrected pCASL time series. This mean image was then brain extracted (BET, FSL) and coregistered to the brain-extracted MPRAGE image using FLIRT with 6 degrees of freedom and trilinear interpolation.

To obtain the ALL90 atlas (in MNI space) in each subject’s native CBF space, the inverse transformations from the previous two coregistrations were applied using the convert_xfm function in FSL. Specifically, the inverse of the MPRAGE-to-MNI transformation and the inverse of the pCASL-to-MPRAGE coregistration were concatenated and applied to the atlas. Finally, for each subject, mean CBF values were extracted within each of the 90 atlas-defined regions, computed in native (CBF) space.

### 2.4. The Tri-Exponential IVIM Model

The IVIM model separates tissue diffusivity from perfusion-related diffusion by analyzing dMRI signal decay across b-values. While the conventional bi-exponential model distinguishes molecular diffusion from pseudodiffusion, it may not fully reflect tissue complexity in areas with heterogeneous cellularity or vascularity [12]. To address these limitations, we employed a tri-exponential IVIM model, which introduces an additional compartment to account for fast-free diffusion, therefore enabling a more nuanced representation of multiple diffusion processes within brain tissue. The tri-exponential IVIM model can be expressed mathematically as follows [18]:

(1) $$\frac{S_{\left(b\right)}}{S_{0}}=\left[F_{p}\cdot exp\left(-{bD}_{p}\right)+F_{f}\cdot exp\left(-{bD}_{f}\right)+F_{s}\cdot exp\left(-{bD}_{s}\right)\right]$$

where *S_b_* represents the signal intensity at a given b-value and *S_0_* is the signal without diffusion weighting. The parameters *F_p_*, *F_f_*, and *F_s_* denote the signal fractions of the three diffusion compartments, and *D_p_*, *D_f_*, and *D_s_* represent their corresponding diffusion coefficients. Specifically, *D_p_* typically reflects perfusion-related diffusion (e.g., microvascular flow), *D_f_* captures fast, free-like diffusion, potentially related to interstitial or extracellular water, and *D_s_* corresponds to slow, restricted diffusion associated with true molecular diffusion within tissue structures.

To enhance the accuracy and robustness of parameter estimation, a two-step fitting procedure was employed. Because the contributions of perfusion and fast-free diffusion become negligible at high b-values, *D_s_* was first estimated using only data with b-values ≥ 1000 s/mm^2^, according to a simplified mono-exponential model:

(2) $$S=S_{0}\cdot exp\left(-{bD}_{s}\right);\;\;for\;b\geq 1000\;s/{mm}^{2}$$

In the second step, the diffusion coefficient *D_f_* was fixed at 3.0 × 10^−3^ mm^2^/s, corresponding to the known diffusivity of free water at body temperature [33]. While this value is based on prior literature, individual variations in tissue microenvironment could affect parameter estimates, potentially influencing the sensitivity of the derived perfusion metrics. The remaining parameters were then estimated by fitting the full tri-exponential model across all b-values (Equation (1)).

After converting the dMRI DICOM images to NIfTI, MRtrix3 (version 3.0.4-145) [34] and FSL (version 6.0.7.16) [35] were used for data pre-processing. The preprocessing pipeline included dMRI denoising using dwidenoise (MRtrix3) [36], EPI distortion correction, eddy current correction, and motion correction (FSL) [37].

Brain extraction was performed on the b0 images using dwi2mask (MRtrix3) [38]. All brain-extracted b0 images were then linearly coregistered to the MNI standard space using FLIRT. Similarly to the pCASL data, the inverted coregistration matrices created from the coregistration between the brain extracted b0 images and the MNI was used to convert the ALL90 atlas into each subject’s native dMRI space.

The tri-exponential IVIM parameter maps were computed using a custom Python (version 3.13.2) script implementing the Levenberg–Marquardt algorithm (curve_fit, SciPy). Parameter estimation was constrained within physiologically plausible bounds: *D_s_* was estimated within 0.00001–0.003 mm^2^/s (initial value 0.001 mm^2^/s), *D_p_* within 0.003–0.2 mm^2^/s (initial value 0.01 mm^2^/s), and *D_f_* was fixed at 0.003 mm^2^/s to enhance model stability. Signal fractions (*F_p_*, *F_f_*, *F_s_*) were constrained to be non-negative and to sum to 1.

Voxels outside the brain mask or affected by signal dropouts were excluded prior to model fitting. All fits were visually inspected to confirm convergence and the absence of non-physical parameter values (e.g., negative diffusion coefficients or signal fractions). Voxel-wise fitting quality was further evaluated through inspection of residual error maps and representative signal–fit curves across tissue types. Although no explicit voxel rejection based on R^2^ thresholds was applied, subsequent analyses were performed only on parameter maps that passed these visual and quantitative quality assessments, ensuring robust and reproducible estimates across participants.

### 2.5. Multimodal Canonical Correlation Analysis with Joint Independent Component Analysis (mCCA+jICA)

In this study, mCCA+jICA [26,27] was employed to integrate two complementary quantitative MRI modalities: CBF maps derived from pCASL and parametric maps from the tri-exponential IVIM model (*D_s_, F_p_*, *F_s_*, and *F_f_*). The goals of this analysis were (1) to identify joint spatial components that distinguish between HCs and individuals with mTBI and (2) to explore associations between imaging-derived components and clinical outcomes, specifically the GOS-E scores in the mTBI group.

In the mCCA stage, canonical variates were computed separately for each modality by maximizing the inter-subject correlations across modalities. These variates, representing maximally correlated projections across subjects, were concatenated across modalities to form a single multimodal feature matrix. This joint matrix was then decomposed using jICA, which assumes that the variance across modalities arises from a set of statistically independent latent sources. This fusion approach yields a set of spatially independent components (ICs), with corresponding subject loadings that are shared across modalities.

**Figure 1:**
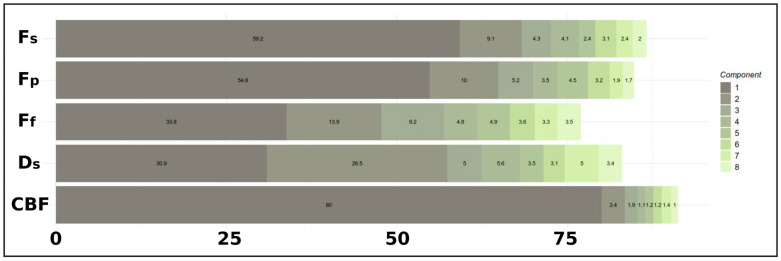
Average variance explained (AVE) by RGCCA components across imaging modalities. Bar plots show the percentage of variance explained by each component (color-coded) in each imaging feature set: *F_s_*, *F_p_*, *F_f_, D_s_* (IVIM), and CBF (ASL). Each segment represents a component’s contribution to the Average Variance Explained (AVE) in that modality. Based on the cumulative AVE across modalities, we selected three components for the mCCA+jICA analysis, as they captured most of the variance while maintaining a simple model structure.

To select the number of components per modality block in a parsimonious and data-driven manner, an initial Regularized Generalized Canonical Correlation Analysis (RGCCA) was performed using the RGCCA package (version 3.0.3) in R (version 4.5.0). A relatively large number of components (e.g., 8) was initially extracted, and the Average Variance Explained (AVE) was calculated for each component across all blocks (Figure 1). Specifically, the outer AVE, which reflects the variance within each modality block explained by its associated canonical variate, was used to assess the contribution of each component. The AVE profile was visualized through cumulative and component-wise plots to identify the point of diminishing returns, thereby guiding the selection of an optimal number of components that balanced explanatory power and model simplicity.

This multimodal fusion framework enables the detection of spatially linked patterns of covariation across imaging modalities, facilitating the identification of modality-concordant (joint) and modality-specific (unique) brain changes.

### 2.6. Statistical Analyses

In this study, sex was based on biological classification at birth, as relevant to brain anatomy. Gender identity was not considered in this analysis.

Table 1 summarizes all demographic and clinical characteristics for each group, including age, sex, GOS-E, mean days from mTBI to MRI exam, and motion (in mm) during pCASL and IVIM acquisitions, presented as means (±standard deviation). Differences in sex distribution were evaluated using a chi-squared test (*X*^2^). Age and motion during pCASL and IVIM acquisitions were assessed for normality via the Shapiro–Wilk test, with group comparisons performed using a Wilcoxon rank-sum test.

To evaluate the test–retest reliability of all parameters, we computed the intraclass correlation coefficient (ICC) [39] for each parameter in a subset of 11 HCs. Voxel-wise ICC values were estimated using a two-way mixed-effects model for absolute agreement (ICC(3,1)), which is suitable for repeated measurements within the same participants under fixed raters. ICC maps were then generated to visualize the spatial distribution of reliability across the brain.

For the mCCA+jICA analysis, input data consisted of region-of-interest (ROI) average values extracted from CBF and tri-exponential IVIM maps using the ALL90 atlas in each subject’s native space. Prior to analysis, each imaging metric was reshaped into a subject × ROI matrix and z-scored across subjects. For group comparisons, five modality-specific matrices (CBF, *F_p_*, *F_f_*, *F_s_*, *D_s_*) were used, while for GOS-E correlation analyses, a sixth matrix containing GOS-E scores was included. All matrices were entered into RGCCA with full inter-block connectivity and regularization parameters (τ) set to 1. Based on the AVE profiles (see Figure 1), three canonical components per block were extracted. The resulting canonical variates were concatenated and decomposed using jICA, via the icafast function from the ica package in R, yielding statistically independent components and subject-specific loading scores.

To assess group differences, two-sample *t*-tests were performed on IC scores between HCs and mTBI patients. Statistical significance was evaluated at *p* < 0.05 (uncorrected) and *p*-FDR < 0.05 (corrected). To further interpret the regional contributions to significant components, Spearman correlation coefficients were computed between IC scores and z-scored ROI values within each modality. For each significant IC, the top five contributing ROIs were identified based on the highest absolute correlation values. Finally, voxel-wise comparisons between HC and mTBI groups were conducted in R within these top ROIs, using two-sample *t*-tests and FDR correction (*p*-FDR < 0.05) to localize subregional effects.

To study the relationship between imaging-derived independent components and clinical outcomes, we conducted mCCA+jICA, integrating IVIM and ASL imaging parameters with GOS-E scores. As in the previous analysis comparing HC and mTBI subjects, we selected the first three principal components per modality block, resulting in 15 ICs. The imaging data blocks included five quantitative parameters: *D_s_*, *F_p_*, *F_f_*, *F_s_*, and CBF from ASL. GOS-E was included as an additional behavioral/clinical block. Component scores were extracted per subject for each IC, and Spearman correlation coefficients were computed between IC scores and individual GOS-E scores to identify components associated with functional outcome. Additionally, correlations between GOS-E score and IVIM/ASL metrics within the AAL90 ROIs were analyzed by Spearman’s correlation coefficient.

## 3. Results

There were no significant group differences observed in sex or age (*X*^2^ = 0.029, *p* = 0.864; Wilcoxon-W = 265, *p* = 0.371, respectively). Within the mTBI group, the mean GOS-E score was 6.4 ± 1.1, and the average time from head injury to MRI examination was 10.85 ± 2.81 days. Furthermore, motion parameters obtained from pCASL and dMRI, including both absolute and relative motion, revealed no significant differences between groups. A comprehensive summary of these results and statistical analyses can be found in Table 1.

### 3.1. Test–Retest Reliability

To evaluate test–retest reliability, ICCs were calculated in a subset of 11 HCs for IVIM-derived maps and ASL-derived CBF (Supplementary Figure S1). Reliability varied across parameters, with *D_s_* showing the highest ICC (0.77 ± 0.19), followed by *F_p_* (0.71 ± 0.20), *F_s_* (0.69 ± 0.21), and CBF (0.66 ± 0.26). The lowest reliability was observed for *F_f_* (0.56 ± 0.24).

### 3.2. Selection of RGCCA Components Based on AVE

The AVE profile was examined to select the number of components for the RGCCA model (Figure 1). The first component explained a substantial portion of the variance across blocks (mean outer AVE ≈ 51.7%), with particularly high contributions for CBF (80.0%), *F_s_* (59.2%), and *F_p_* (54.8%). The second component added considerable variance (mean outer AVE ≈ 12.6%), notably for the *D_s_* (26.5%), *F_f_* (13.9%), and *F_p_* (10.0%) blocks. The third component further contributed moderately. Components 4 through 8 demonstrated progressively smaller increments in the explained variance across most blocks, indicating diminishing returns. Based on this AVE profile, the first three components per block were selected for the final RGCCA model.

### 3.3. mCCA+jICA: HC vs. mTBI

**Figure 2:**
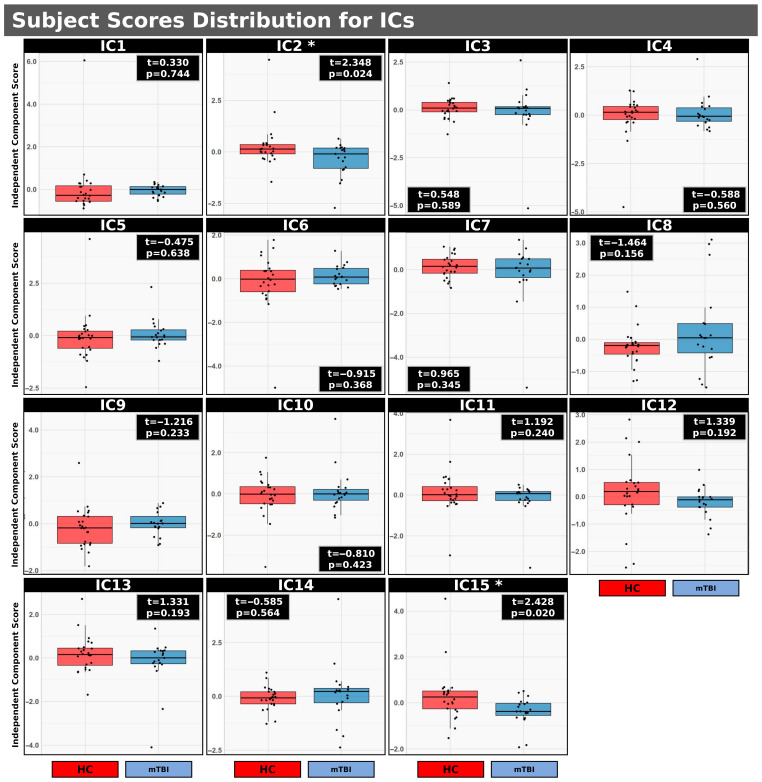
Distribution of subject scores for 15 Independent Components (ICs) derived from a mCCA+jICA. Boxplots showing the distribution of subject scores for 15 Independent Components (ICs) derived from a mCCA+jICA comparing HC and individuals with mTBI. Each IC represents a spatially independent source identified across multimodal imaging data, derived using the first three components per block in a regularized generalized CCA (RGCCA) model. The five imaging parameters used in the analysis were: *F_s_, F_p_, F_f_, D_s_* for IVIM and CBF for ASL. * Significant group differences were observed for IC2 and IC15 (*p* < 0.05; uncorrected). No significant differences were found after FDR correction.

Figure 2 illustrates the distribution of subject scores for 15 ICs resulting from mCCA+jICA comparing HC and individuals with mTBI. Each IC represents a spatially independent source identified across the integrated imaging modalities. The initial three components per block for five imaging parameters (*D_s_, F_p_, F_s_, F_f_* for IVIM and CBF for ASL) were included in the analysis. Significant uncorrected group differences (*p* < 0.05) were observed specifically for IC2 (t = 2.348, *p* = 0.024) and IC15 (t = 2.428, *p* = 0.020). In both IC2 and IC15, the distribution of subject scores was higher in the HC group compared to the mTBI group (IC2: <HC> = 0.041, <mTBI> = −0.052; IC15: <HC> = 0.298, <mTBI> = −0.377). However, while uncorrected significant differences were observed for IC2 and IC15, these differences did not persist after applying FDR correction. These findings suggest that distinct patterns of multimodal brain activity, as characterized by IC2 and IC15, may serve as potential discriminators between individuals with mTBI and HCs. A complete summary of group comparisons for all 15 ICs identified via mCCA+jICA, comparing HC and individuals with mTBI, is presented in Table 2.

**Figure 3:**
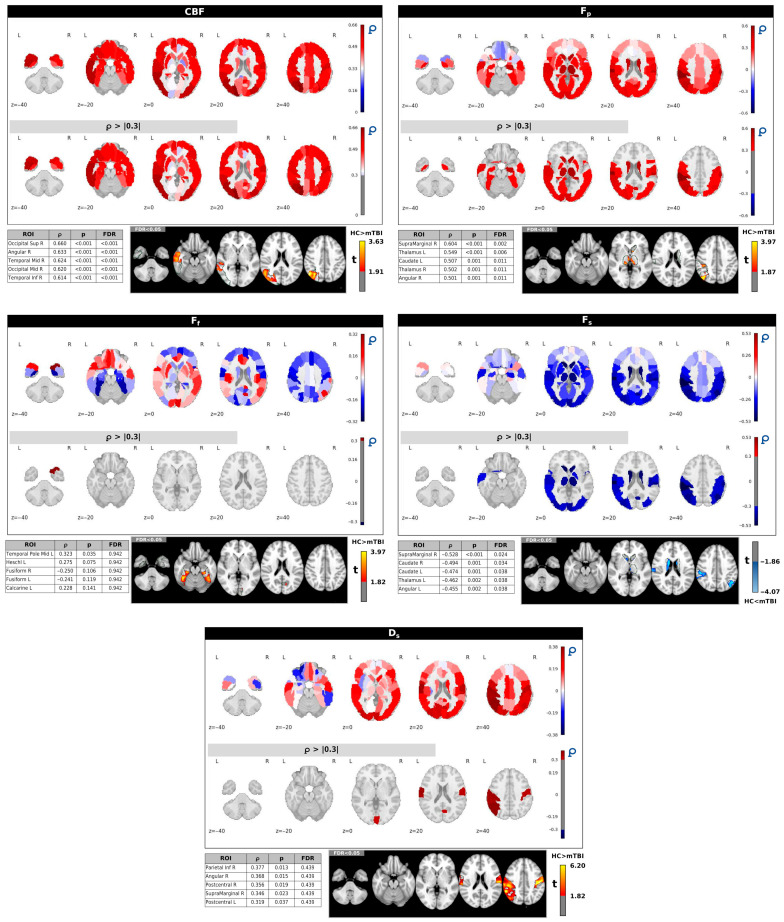
Correlation maps between IC2 and regional brain measures from CBF, *F_p_, F_s_, F_f_,* and *D_s_* across AAL90 regions. The bottom panels show the top five regions with the highest Spearman’s correlation coefficient |ρ| values with *p* and FDR values and corresponding voxel-wise *t*-tests between HC and mTBI subjects.

Figure 3 illustrates the spatial correlation patterns between IC2, which was identified as significantly different between HC and individuals with mTBI (*p* = 0.024), and the ASL/IVIM metrics. These correlations were computed across regions defined by the AAL90 atlas. Each panel presents whole-brain correlation maps for a specific imaging metric, with the corresponding thresholded maps (Spearman’s correlation coefficient ∣ρ∣ > 0.3). The five regions with the strongest correlations (highest ∣ρ∣ values) are provided for each metric, along with voxel-wise *t*-test results comparing HC and mTBI groups within those regions.

More specifically, IC2 exhibited significant positive correlations with CBF and *F_p_* in several brain regions. For CBF, strong positive correlations were observed in regions such as the right superior/middle occipital gyrus and right angular gyrus, with all listed regions remaining significant after FDR correction. Similarly, *F_p_* showed significant positive correlations with IC2 in regions including the thalamus and left caudate (all FDR-corrected *p* < 0.05). These positive correlations align with the higher IC2 scores observed in HCs (Figure 2), suggesting that higher perfusion (CBF) and perfusion fraction (*F_p_*) in these regions are associated with the healthy brain state.

In contrast, *F_s_* displayed primarily negative correlations with IC2, particularly in subcortical and frontal areas such as the thalamus and caudate (all FDR-corrected *p* < 0.05). This negative relationship implies that higher IC2 scores (characteristic of HCs) are associated with lower *F_s_* in these regions. Conversely, mTBI subjects, with lower IC2 scores, tend to exhibit higher *F_s_* in these areas, which may indicate vascular injury, microvascular dysfunction, or hypoperfusion.

IC2 also showed positive correlations with *D_s_*, mainly in the right inferior parietal lobule, right angular gyrus, and postcentral gyrus. However, these correlations did not remain significant after FDR correction. No significant correlations were found for *F_f_* at an FDR-corrected *p* < 0.05.

The voxel-based *t*-tests, presented for the top five correlated regions for each metric, further corroborate these observed group differences, indicating significant differences between groups within these specific brain areas. Supplementary Table S1 shows the complete correlations, *p* and FDR values for all ROIs included in the AAL90 atlas.

**Figure 4:**
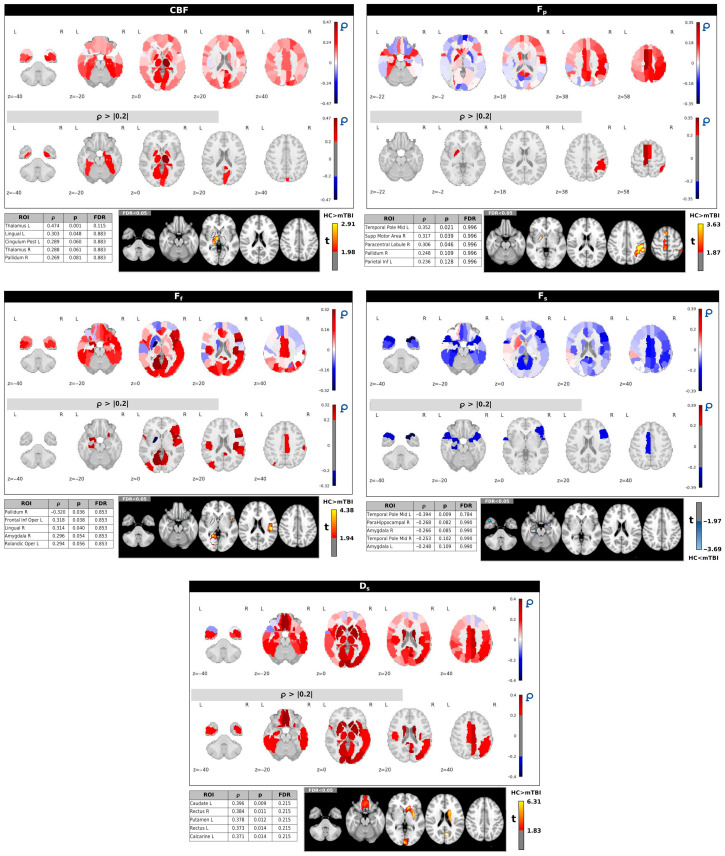
Correlation maps between IC15 and regional brain measures from CBF, *D_s_, F_p_, F_s_, F_f_* across AAL90 regions. The bottom panels show the top five regions with the highest Spearman’s correlation coefficient |ρ| values with *p* and FDR values and corresponding voxel-wise *t*-tests between HC and mTBI subjects.

Figure 4 shows the spatial correlation patterns between IC15 (*p* = 0.020) and the ASL/IVIM metrics. These correlations were computed across all 90 regions of the AAL90 atlas. Each panel presents whole-brain correlation maps for a specific imaging metric, alongside corresponding thresholded maps (∣ρ∣ > 0.2). Consistent with Figure 3, accompanying tables list the five regions demonstrating the strongest correlations (highest ∣ρ∣ values) for each metric, along with voxel-wise *t*-test results comparing HC and mTBI groups within those top regions.

IC15 generally showed positive correlations with CBF across many brain areas, particularly in structures such as the thalamus, lingual gyrus, and right pallidum. While these regions often exhibited higher CBF in HCs compared to mTBI subjects using voxel-based analysis, the positive correlations with IC15 in the left thalamus and left lingual gyrus (*p* < 0.05) did not remain significant after FDR correction.

For *F_p_* and *F_f_*, IC15 displayed both negative and positive correlations. Given that the HC group generally had higher *F_p_* and *F_f_* values compared to the mTBI group, these varied correlations suggest complex relationships between IC15 and these perfusion parameters depending on the specific brain region.

F_s_ primarily exhibited negative correlations with IC15, notably in the left temporal pole and amygdala. This negative relationship indicates that areas with higher *F_s_* (which were observed in mTBI subjects compared to HCs) are associated with lower IC15 scores, aligning with the pattern that lower IC15 scores are characteristic of the mTB group.

Finally, *D_s_* showed positive correlations with IC15 in areas with high ∣ρ∣ values, including the left caudate, left putamen, and rectus gyrus (*p* < 0.05). However, similar to CBF, these correlations for *D_s_* did not remain significant after FDR correction.

The voxel-based *t*-tests, presented for the top five correlated regions for each metric, further support the observed group differences within these specific brain areas. A complete summary of correlations, *p*-values, and FDR values for all AAL90 ROIs is provided in Supplementary Table S2.

### 3.4. mCCA+jICA: Correlation with GOS-E

**Figure 5:**
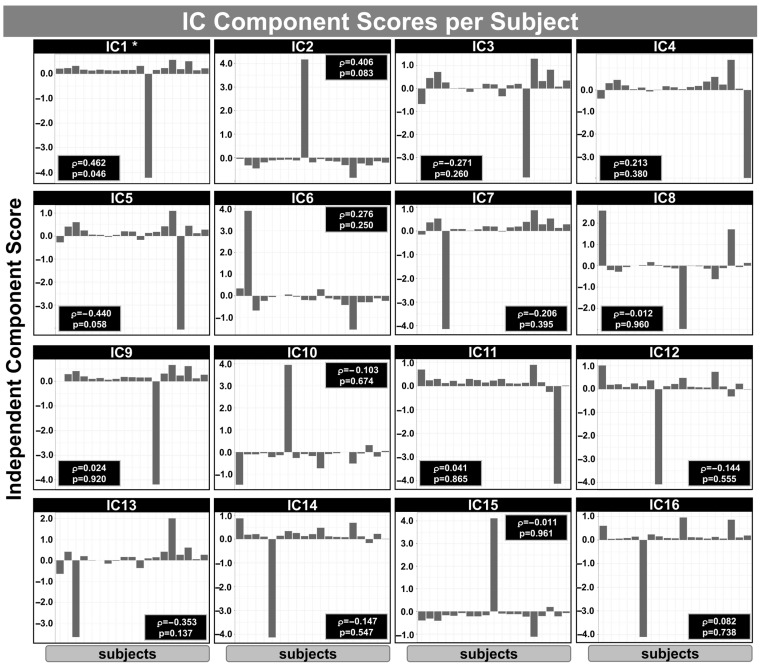
Independent component (IC) scores per subject obtained from mCCA+jICA integrating *D_s_, F_p_, F_s_, F_f_* (from IVIM), pCASL-derived CBF, and GOS-E scores in the mTBI group. Each panel (IC1–IC16) displays the subject-wise loading values for one IC, representing the degree to which each subject expresses the corresponding multimodal spatial pattern. Spearman’s correlation coefficients (ρ) and uncorrected *p*-values indicate the association between each component and GOS-E scores. IC1 showed a significant positive correlation with GOS-E (ρ = 0.462, *p* = 0.046), suggesting that higher expression of this component may be associated with better functional outcome following mTBI. No other components reached statistical significance. Only 16 components were extracted (rather than 18) due to collinearity or redundancy among input variables. * significant at *p* < 0.05 uncorrected.

Figure 5 shows the IC subject scores obtained from mCCA+jICA for the relationship between IVIM/ASL metrics and GOS-E scores within the mTBI group. The analysis was based on the first three components per block using an RGCCA model integrating *D_s_, F_p_, F_s_, F_f_* (from the IVIM model), pCASL-derived CBF, and GOS-E scores. Each panel (IC1–IC16) displays the subject-wise loading values for one IC, representing the degree to which each subject expresses the corresponding multimodal spatial pattern. Spearman’s correlation coefficients (ρ) and uncorrected *p*-values indicate the association between each component’s subject scores and GOS-E scores. Notably, IC1 showed a significant positive correlation with GOS-E (ρ = 0.462, *p* = 0.046), suggesting that higher expression of this particular component in mTBI subjects may be associated with better functional outcome following mTBI. No other components reached statistical significance (*p* < 0.05). It is important to note that 16 components were extracted from this analysis, rather than 18, due to collinearity or redundancy among the input variables. Table 3 shows the summary of the GOS-E correlation inside each IC identified using the mCCA+jICA method.

**Figure 6:**
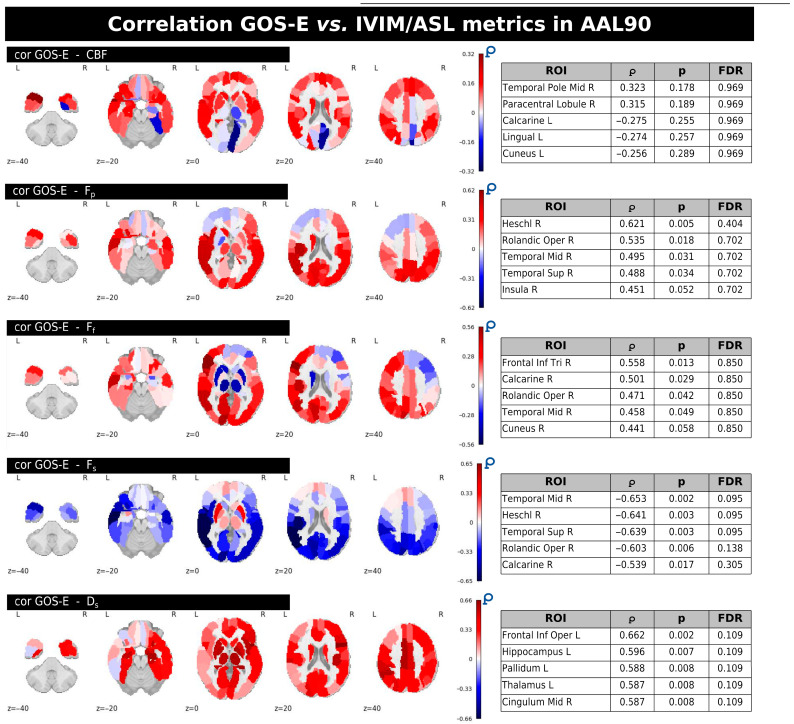
Correlation maps for the ALL90 atlas showing associations between GOS-E and individual IVIM/ASL metrics. The tables list the top five AAL90 regions with the highest correlation values for each metric, along with raw and FDR-corrected *p*-values.

Figure 6 displays the regional correlations between GOS-E scores and individual ASL/IVIM metrics within the mTBI group. Each panel provides whole-brain correlation maps along with tables listing the five regions from the AAL90 atlas that exhibited the strongest correlation magnitudes (∣ρ∣ values). These tables also include the corresponding uncorrected and FDR-corrected *p*-values. While various brain regions showed uncorrected correlations (*p* < 0.05) with GOS-E for several metrics, no significant correlations persisted after applying FDR correction across any of the IVIM/ASL parameters.

In general, CBF predominantly showed both positive and negative correlations with GOS-E, notably in regions like the right middle temporal gyrus and the right paracentral lobule, left lingual and cuneus, but none of these reached significance after FDR correction.

*F_p_* and *F_f_* largely exhibited positive correlations with GOS-E in several regions, including the right Heschl gyrus (for *F_p_*) and right inferior frontal gyrus, triangular part, right calcarine, and right cuneus (for *F_f_*). *F_s_* demonstrated mainly negative correlations with GOS-E, with stronger associations observed in areas such as the right middle temporal gyrus and right Heschl gyrus. Finally, *D_s_* showed positive correlations with GOS-E in regions including the left frontal inferior operculum, left hippocampus, and left pallidum; however, similar to the other metrics, these correlations were not significant following FDR correction. A complete summary of GOS-E correlations with the IVIM/ASL metrics inside the AAL90 ROIs with uncorrected and FDR-corrected *p*-values is provided in Supplementary Table S3.

## 4. Discussion

mTBI is a complex pathophysiological condition typically caused by biomechanical forces and often lacking detectable structural abnormalities on conventional imaging. As a result, advanced MRI techniques have become crucial for investigating the subtle neurobiological changes underlying post-concussive symptoms. Among these techniques, dMRI has been widely employed to assess microstructural white matter abnormalities associated with mTBI. In particular, DTI has revealed alterations in concussed individuals in key metrics such as fractional anisotropy and mean diffusivity, providing indirect markers of axonal injury or demyelination [5,40,41,42,43]. In addition, ASL has been used to evaluate CBF changes following mTBI, which may reflect mechanisms such as neurovascular uncoupling, autonomic dysfunction, or neuroinflammation [43,44,45].

To our knowledge, this is the first study to apply the tri-exponential IVIM model to mTBI. By improving separation of diffusion components, allowing the differentiation of fast (vascular), intermediate (free), and slow (restricted) diffusion compartments, this model may enhance sensitivity to subtle microvascular and microstructural changes [18]. Test–retest analysis showed good reliability for IVIM diffusivity parameters (*D_s_* and *F_p_*), while perfusion-related indices (*F_f_* and *F_s_*) and ASL-derived CBF were only moderately reliable. These findings suggest that diffusivity measures might be more robust across sessions, whereas perfusion estimates remain more variable.

We employed the advanced mCCA+jICA method [26,27] to examine shared patterns across IVIM-derived diffusion metrics and ASL-based CBF between HCs and individuals with mTBI. This multivariate, data-driven approach enables the integration of multimodal imaging features and the identification of shared patterns of variance across modalities, potentially capturing mTBI-related effects that are not detectable through unimodal analyses. Among the 15 ICs identified, two components (IC2 and IC15; Figure 2) showed uncorrected significant group differences (*p* < 0.05), with higher subject scores in the HC group. Although these differences did not survive FDR correction, their spatial and metric-level correlations reveal patterns consistent with known cerebrovascular and microstructural pathophysiology.

IC2, which showed a significant group difference (*p* = 0.024, uncorrected), was positively correlated with CBF and *Fp* across several cortical and subcortical regions, including the right superior/middle occipital gyrus, right angular gyrus, thalamus, and left caudate. These associations, which remained significant after FDR correction, suggest that IC2 may reflect patterns of healthy perfusion and vascular integrity, which appear diminished in the mTBI group. IC2 was negatively correlated with Fs in subcortical and frontal areas, indicating that more restricted diffusion, potentially associated with microvascular dysfunction or tissue damage, corresponds to lower IC2 scores in mTBI. IC2 also exhibited positive correlations with *Ds* in regions including the right inferior parietal lobule, angular gyrus, and postcentral gyrus. Although these associations did not survive FDR correction, they suggest that higher IC2 scores in HC may reflect preserved tissue diffusivity. Collectively, these findings provide preliminary evidence of multimodal patterns of cerebrovascular and microstructural status that may be altered in mTBI.

The spatial correlation patterns for IC15, which also showed a group difference (*p* = 0.020, uncorrected), were more variable. IC15 was generally positively correlated with CBF in several regions, including the thalamus, lingual gyrus, and right pallidum. Although these correlations did not remain significant after FDR correction, the overall pattern mirrors the directionality observed in IC2: higher CBF correlates with higher component scores in HCs. *Fs* demonstrated negative correlations with IC15, notably in the left temporal pole and amygdala, suggesting a consistent pattern in which elevated *Fs* may be associated with mTBI-related alterations. Positive correlations between IC15 and *Ds* were also observed in regions such as the left caudate and putamen, though these may represent secondary or non-specific effects.

These results were confirmed by voxel-based analysis within the regions with highest correlations for each metric, thus reinforcing the utility of mCCA+jICA in identifying latent brain patterns that integrate multimodal structural and perfusion information. The consistent group differences in IC2 and IC15, together with their meaningful spatial associations with physiologically relevant parameters, suggest that these components capture biologically plausible signatures of mTBI-related alterations. Importantly, the observed relationships indicate that mTBI is associated with reduced perfusion (lower CBF and *F_p_*) and potentially increased *Fs*, particularly in subcortical regions such as the thalamus and caudate, structures repeatedly implicated in mTBI literature [46,47,48].

Changes in CBF, cerebrovascular reactivity, and blood–brain barrier permeability have been observed in both the acute and chronic phases of injury following all severities of TBI [49]. Prior studies have reported both increased and decreased CBF following mTBI, likely reflecting temporal dynamics and individual variability. Although some studies have found hyperperfusion in the acute concussion phase [50], hypoperfusion has been more consistently reported, particularly in subacute and chronic stages, and linked to worse outcomes [51,52,53,54]. Significantly lower CBF was previously observed in concussed athletes, which also correlated with clinical assessments [51]; moreover, hyperperfusion has been associated with reduced axonal integrity in middle-aged veterans with history of mild or moderate mTBI [53]. Reduced CBF has been further linked to persistent post-concussive symptoms, including cognitive impairment, fatigue, and sleep problems [54]. These changes likely reflect impaired neurovascular function and metabolic dysregulation in mTBI.

While IVIM has not been previously applied to mTBI, DTI remains widely employed to assess microstructural integrity in mTBI [55], with reduced FA and increased MD interpreted as indicators of axonal injury, myelin damage, or neuroinflammation, which are hallmarks of post-concussion pathology [55]. These changes are frequently observed in regions vulnerable to shearing forces, including the corpus callosum, fronto-temporal white matter tracts, and pathways associated with cognitive function and emotional regulation [56]. DTI-based changes within these tracts collectively point towards diffuse axonal injury as a critical component of mTBI pathophysiology. The presence and extent of these DTI-derived abnormalities have also been shown to correlate with the severity of acute symptoms and, importantly, with the persistence of post-concussive symptoms, including cognitive deficits, fatigue, and headaches [57].

The mCCA+jICA method was employed to explore the relationship between IVIM/ASL metrics and functional outcomes, as measured by GOS-E, within the mTBI group. A significant positive association between IC1 and GOS-E (*p* = 0.046; Figure 5) suggests that this component may reflect cerebral physiological patterns linked to more favorable post-mTBI recovery. Within IC1, IVIM parameters *Fp* and *Ff* exhibited predominantly positive correlations in regions such as the Heschl gyrus and inferior frontal gyrus, potentially reflecting preserved or compensatory perfusion mechanisms that support neural resilience. Similarly, *Ds* showed positive correlations in regions including the hippocampus and inferior frontal operculum, possibly indicating microstructural preservation. ASL-derived CBF exhibited both positive and negative correlations across temporal and occipital cortices, confirming prior findings of heterogeneous CBF changes in acute concussion [51]. These mixed patterns may reflect regional variations in perfusion regulation or metabolic demand following injury. The overall positive relationship between IC1 and GOS-E, encompassing both perfusion- and diffusion-related metrics, may point to preserved neurovascular coupling or effective microvascular adaptation in individual with better recovery trajectories. Although none of the individual IVIM or ASL metrics demonstrated statistically significant associations with GOS-E after FDR correction, the observed uncorrected correlations are consistent with established mTBI pathophysiology and underscores the value of multimodal fusion approaches like mCCA+jICA in capturing distributed, physiologically relevant patterns that may be undetectable through univariate techniques alone.

While most group-level differences did not survive correction for multiple comparisons, several region- and metric-level correlations (e.g., between IC2 and CBF, *Fp*, and *Fs*) remained significant after FDR adjustment, supporting the physiological plausibility of our findings. Importantly, our conclusions are intentionally conservative, reflecting the exploratory nature of this pilot study. The findings demonstrate methodological feasibility and provide preliminary evidence to guide future, larger-scale studies.

This study has several limitations. First, imaging data for the mTBI group were collected at varying time points within a post-injury window of 7 to 15 days. Although this subacute period is clinically relevant for capturing evolving neurophysiological changes, variability in scan timing may have introduced heterogeneity in the imaging results. Individual differences in recovery trajectories during this interval could have influenced both IVIM and ASL measures, potentially masking temporally specific effects. Future research would benefit from a more narrowly defined post-injury timeframe or, ideally, longitudinal imaging to better delineate the temporal progression of perfusion and microstructural changes.

Second, the relatively small sample size of the mTBI cohort limits statistical power and increases the risk of type II errors. Because this was designed as a pilot, hypothesis-generating study, no formal power calculation was performed; rather, our aim was to assess methodological feasibility and generate preliminary effect-size estimates to inform larger trials. Additionally, the mTBI group was heterogeneous: while most participants had clinically diagnosed concussions, two participants presented with intracranial hemorrhages (one due to motor-vehicle accident, one fall-related). This variability reflects the clinical diversity of mTBI presentations encountered in emergency settings but may contribute to additional imaging heterogeneity. All participants were evaluated by a physician prior to enrollment, and inclusion was based on standard clinical criteria for mTBI. Finally, we selected the Glasgow Outcome Scale–Extended (GOS-E) as our outcome measure because it provides a validated, global index of functional recovery that facilitates comparison across studies and injury severities.

Finally, the advanced MRI techniques employed here, including tri-exponential IVIM and pCASL, require specialized hardware and software that may not be widely accessible across all clinical or research centers. This could limit the replicability and generalizability of our findings. Future studies should consider alternative imaging protocols or multi-center collaborations to address these technical limitations and promote broader application of similar research.

Despite these limitations, the use of a multimodal fusion approach offers enhanced sensitivity in small datasets by leveraging shared information across modalities. Together, these limitations highlight the need for larger, more temporally controlled studies to validate and extend the current findings. The major strength of this work lies in its methodological innovation: to our knowledge, this is the first study to apply a tri-exponential IVIM model alongside ASL perfusion metrics within a multimodal data fusion framework using mCCA+jICA to investigate mTBI. This integrative approach may capture complex, distributed physiological patterns that may underlie post-mTBI outcomes, offering preliminary but compelling evidence that IVIM and ASL-derived imaging markers are associated with functional recovery in mTBI.

## 5. Conclusions

This pilot study demonstrates the feasibility of integrating tri-exponential IVIM and ASL MRI metrics within a multimodal data fusion framework to detect subtle cerebral alterations after mTBI. Using mCCA+jICA, we identified joint patterns of diffusion and perfusion abnormalities that distinguished individuals with mTBI from HCs and related to functional outcomes. These results provide preliminary support for multimodal MRI, combined with advanced integrative analyses, as a potential biomarker of injury and recovery in mTBI.

Importantly, our findings should be interpreted with caution given the small, heterogeneous cohort and the fact that most group differences did not survive correction for multiple comparisons. The associations with clinical outcomes, while biologically plausible, were modest and require replication. Future multicenter studies with larger, more homogeneous samples are needed to validate these observations and to further explore their diagnostic and prognostic utility.

## Figures and Tables

**Table 1:** Group differences in sex distribution, age, and MRI motion parameters during pseudo-continuous arterial spin labeling (pCASL) and intravoxel incoherent motion (IVIM) sequences are summarized.

Group	# (F)	Age (SD)	GOS-E	Mean Days MRI Exam After Head Injury
HC	24 (12)	29.4 (6.2)	—	—
mTBI	19 (10)	28 (7.7)	6.4 (1.1)	10.85 (2.81)
Sex:				
*X*^2^ test	*X*^2^ = 0.029; *p* = 0.864			
Age:				
Shapiro–Wilk normality test				
HC		W = 0.912; *p* = 0.039		
Concussion		W = 0.880; *p* = 0.022		
Wilcoxon rank-sum		W = 265; *p* = 0.371		
Motion during MRI pCASL	ABS (mm)	REL (mm)		
HC	0.169 (0.198)	0.059 (0.079)		
mTBI	0.151 (0.133)	0.056 (0.062)		
Shapiro–Wilk normality test				
HC	W = 0.678; *p* < 0.001	W = 0.668; *p* < 0.001		
Concussion	W = 0.739; *p* < 0.001	W = 0.762; *p* < 0.001		
Wilcoxon rank-sum	W = 183; *p* = 0.280	W = 211; *p* = 0.690		
Motion during MRI IVIM	ABS (mm)	REL (mm)		
HC	0.761 (0.304)	0.270 (0.039)		
mTBI	0.823 (0.343)	0.305 (0.094)		
Shapiro–Wilk normality test				
HC	W = 0.831; *p* = 0.001	W = 0.906; *p* = 0.029		
Concussion	W = 0.917; *p* = 0.097	W = 0.755; *p* < 0.001		
Wilcoxon rank-sum	W = 197; *p* = 0.460	W = 190; *p* = 0.363		

Sex distribution was compared using a chi-squared test (*X*^2^). Age distributions were assessed for normality using the Shapiro–Wilk test and compared using a Wilcoxon rank-sum test. MRI motion metrics are shown as absolute (ABS, in mm) and relative (REL, in mm) displacements, with corresponding means and standard deviations. Shapiro–Wilk tests assessed normality of motion data in each group, and group comparisons were performed using Wilcoxon rank-sum tests. For the mTBI group, Glasgow Outcome Scale—Extended (GOS-E) scores and mean days between concussion and MRI examination are also reported.

**Table 2:** Summary of group comparisons for 15 Independent Components (ICs) identified using mCCA+jICA comparing HC and individuals with mTBI.

IC	t	*p*	FDR	<HC>	<mTBI>	Conf. Interval
IC1	0.330	0.744	0.744	0.041	−0.052	(−0.486; 0.672)
IC2	2.348	0.024 *	0.180	0.298	−0.376	(0.094; 1.254)
IC3	0.548	0.589	0.680	0.083	−0.105	(−0.523; 0.899)
IC4	−0.588	0.560	0.680	−0.078	0.099	(−0.786; 0.432)
IC5	−0.475	0.638	0.684	−0.062	0.078	(−0.739; 0.458)
IC6	−0.915	0.368	0.613	−0.115	0.145	(−0.841; 0.321)
IC7	0.965	0.345	0.613	0.145	−0.183	(−0.377; 1.033)
IC8	−1.464	0.156	0.514	−0.214	0.270	(−1.165; 0.198)
IC9	−1.216	0.233	0.514	−0.171	0.216	(−1.036; 0.261)
IC10	−0.810	0.423	0.635	−0.112	0.141	(−0.887; 0.38)
IC11	1.192	0.240	0.514	0.158	−0.200	(−0.249; 0.965)
IC12	1.261	0.216	0.514	0.159	−0.201	(−0.221; 0.94)
IC13	1.331	0.193	0.514	0.188	−0.237	(−0.226; 1.076)
IC14	−0.585	0.564	0.680	−0.089	0.112	(−0.91; 0.509)
IC15	2.428	0.020 *	0.180	0.298	−0.377	(0.112; 1.238)

The analysis was based on the first three components per block using an RGCCA model, incorporating five imaging parameters: *Fs, Fp, Ff, Ds* for IVIM and CBF for ASL. The table includes t-statistics, *p*-values (uncorrected), FDR-corrected *p*-values, mean component scores for each group, and 95% confidence intervals for the difference in means. * Significant group differences were observed for IC2 and IC15 (*p* < 0.05; uncorrected). No significant differences were found after FDR correction.

**Table 3:** Summary of the GOS-E correlation inside each Independent Components (ICs) identified using mCCA+jICA.

IC	ρ	*p*	FDR
IC1	0.463	0.046 *	0.447
IC2	0.407	0.084	0.447
IC3	−0.272	0.261	0.695
IC4	0.214	0.380	0.790
IC5	−0.441	0.059	0.447
IC6	0.277	0.251	0.695
IC7	−0.207	0.395	0.790
IC8	−0.012	0.961	0.961
IC9	0.025	0.920	0.961
IC10	−0.103	0.674	0.961
IC11	0.042	0.866	0.961
IC12	−0.144	0.556	0.889
IC13	−0.353	0.138	0.552
IC14	−0.147	0.548	0.889
IC15	−0.012	0.961	0.961
IC16	0.082	0.738	0.961

The analysis was based on the first three components per block using an RGCCA model, incorporating five imaging parameters: *F_s_, F_p_, F_f_, D_s_* for IVIM model, CBF for ASL, and GOS-E score for the only mTBI group. The table includes Spearman coefficient (ρ), *p*-values (uncorrected), and FDR correction. * Significant group differences were observed for IC1 (*p* < 0.05; uncorrected). No significant differences were found after FDR correction.

## Data Availability

The data presented in this study are not available on request from the corresponding author due to privacy restrictions.

## Supplementary Materials

The following supporting information can be downloaded at: https://www.mdpi.com/article/10.3390/neurosci6040123/s1, Figure S1: Test–retest reliability of IVIM-derived parameters and ASL-derived CBF in 11 healthy controls; Table S1: AAL90 complete correlations, *p*-values, and FDR for IC2; Table S2: AAL90 complete correlations, *p*-values, and FDR for IC15; Table S3: Complete correlations between GOS-E and IVIM/ASL metrics inside the ROIs of the AAL90 atlas.
