# Supplementary material for: Multimodal Canonical Correlation Analysis with Joint Independent Component Analysis (mCCA+jICA) of IVIM and ASL MRI Reveals Perfusion and Diffusion Abnormalities in mTBI—A Pilot Study
Source: NeuroSci. 2025 Dec 3;6(4):123. doi: 10.3390/neurosci6040123 (PMC12735935; doi:10.3390/neurosci6040123)
Supplement: Supplementary file 1 [file neurosci-06-00123-s001.zip › neurosci-3823039-supplementary.pdf]

Table S1. AAL90 complete correlations, p-values, and FDR for IC2.

	IC2														
	**CBF**			**Fp**			**Ff**			**Fs**			**Ds**		
**ROI**	ϱ	**p**	**FDR**	ϱ	**p**	**FDR**	ϱ	**p**	**FDR**	ϱ	**p**	**FDR**	ϱ	**p**	**FDR**
Amygdala L	0.484	0.001	0.002	0.234	0.130	0.244	-0.088	0.574	0.942	-0.093	0.554	0.813	-0.057	0.718	0.972
Amygdala R	0.501	0.001	0.001	0.048	0.761	0.848	-0.029	0.855	0.942	-0.006	0.971	0.983	-0.139	0.373	0.865
Angular L	0.507	0.001	0.001	0.419	0.005	0.030	0.207	0.184	0.942	-0.455	0.002	0.038	0.298	0.052	0.439
Angular R	0.633	<0.001	<0.001	0.501	0.001	0.011	-0.022	0.887	0.942	-0.437	0.003	0.043	0.368	0.015	0.439
Calcarine L	0.375	0.013	0.015	0.444	0.003	0.025	0.228	0.141	0.942	-0.421	0.005	0.054	0.301	0.050	0.439
Calcarine R	0.290	0.059	0.061	0.435	0.004	0.025	0.137	0.382	0.942	-0.341	0.025	0.123	0.280	0.069	0.439
Caudate L	0.435	0.004	0.004	0.507	0.001	0.011	-0.088	0.577	0.942	-0.474	0.001	0.038	0.180	0.248	0.752
Caudate R	0.432	0.004	0.004	0.484	0.001	0.011	-0.072	0.645	0.942	-0.494	0.001	0.034	0.110	0.481	0.932
Cingulum Ant L	0.301	0.050	0.052	-0.023	0.884	0.949	0.116	0.457	0.942	-0.037	0.812	0.928	0.112	0.476	0.932
Cingulum Ant R	0.450	0.002	0.003	-0.002	0.988	0.988	0.157	0.316	0.942	-0.082	0.603	0.824	0.061	0.696	0.957
Cingulum Mid L	0.472	0.001	0.002	0.155	0.320	0.477	-0.006	0.969	0.969	-0.125	0.424	0.761	0.150	0.336	0.820
Cingulum Mid R	0.470	0.001	0.002	0.149	0.339	0.497	-0.022	0.889	0.942	-0.106	0.497	0.798	0.183	0.240	0.752
Cingulum Post L	0.445	0.003	0.003	0.111	0.478	0.613	0.178	0.254	0.942	-0.182	0.242	0.463	0.204	0.189	0.723
Cingulum Post R	0.248	0.109	0.109	0.071	0.651	0.796	0.036	0.820	0.942	-0.074	0.637	0.824	0.119	0.447	0.932
Cuneus L	0.455	0.002	0.003	0.296	0.054	0.119	-0.044	0.780	0.942	-0.250	0.106	0.267	0.220	0.157	0.690
Cuneus R	0.425	0.005	0.005	0.333	0.029	0.089	-0.035	0.825	0.942	-0.252	0.103	0.267	0.290	0.059	0.439
Frontal Inf Oper L	0.508	0.001	0.001	0.302	0.049	0.119	-0.200	0.198	0.942	-0.233	0.132	0.314	0.225	0.147	0.681
Frontal Inf Oper R	0.502	0.001	0.001	0.296	0.054	0.119	-0.026	0.869	0.942	-0.252	0.103	0.267	0.108	0.490	0.932
Frontal Inf Orb L	0.507	0.001	0.001	0.054	0.733	0.830	0.018	0.910	0.942	-0.107	0.494	0.798	-0.043	0.785	0.978
Frontal Inf Orb R	0.569	<0.001	<0.001	0.005	0.976	0.988	0.089	0.572	0.942	-0.093	0.554	0.813	-0.169	0.279	0.768
Frontal Inf Tri L	0.563	<0.001	<0.001	0.187	0.229	0.380	-0.046	0.768	0.942	-0.094	0.547	0.813	0.187	0.231	0.752
Frontal Inf Tri R	0.558	<0.001	<0.001	0.178	0.254	0.407	0.021	0.894	0.942	-0.136	0.386	0.707	0.166	0.289	0.770
Frontal Med Orb L	0.494	0.001	0.001	-0.018	0.911	0.966	0.032	0.838	0.942	-0.009	0.952	0.975	0.032	0.840	0.998
Frontal Med Orb R	0.602	<0.001	<0.001	-0.011	0.942	0.987	0.190	0.223	0.942	-0.097	0.536	0.813	0.018	0.908	0.998
Frontal Mid L	0.548	<0.001	0.001	0.117	0.454	0.606	-0.101	0.518	0.942	-0.050	0.751	0.905	0.092	0.559	0.932
Frontal Mid Orb L	0.545	<0.001	0.001	0.004	0.981	0.988	-0.022	0.888	0.942	-0.057	0.716	0.900	-0.176	0.258	0.752
Frontal Mid Orb R	0.532	<0.001	0.001	-0.056	0.719	0.830	0.032	0.837	0.942	-0.054	0.730	0.905	-0.282	0.067	0.439
Frontal Mid R	0.533	<0.001	0.001	0.130	0.406	0.566	-0.118	0.452	0.942	-0.078	0.619	0.824	0.103	0.512	0.932
Frontal Sup L	0.520	<0.001	0.001	0.106	0.498	0.626	-0.117	0.456	0.942	-0.082	0.603	0.824	0.076	0.626	0.932
Frontal Sup Medial L	0.489	0.001	0.001	0.034	0.827	0.900	-0.184	0.239	0.942	0.024	0.877	0.966	-0.014	0.931	0.998
Frontal Sup Medial R	0.552	<0.001	<0.001	0.034	0.829	0.900	-0.080	0.609	0.942	-0.012	0.941	0.975	-0.009	0.954	0.998
Frontal Sup Orb L	0.479	0.001	0.002	-0.063	0.689	0.830	-0.013	0.936	0.958	-0.045	0.776	0.923	0.039	0.806	0.981
Frontal Sup Orb R	0.521	<0.001	0.001	-0.120	0.442	0.599	0.120	0.444	0.942	-0.063	0.688	0.877	-0.091	0.561	0.932
Frontal Sup R	0.447	0.003	0.003	0.120	0.442	0.599	-0.091	0.560	0.942	-0.109	0.486	0.798	0.042	0.789	0.978
Fusiform L	0.435	0.004	0.004	0.340	0.026	0.081	-0.241	0.119	0.942	-0.019	0.902	0.975	0.053	0.734	0.978
Fusiform R	0.479	0.001	0.002	0.394	0.009	0.040	-0.250	0.106	0.942	-0.077	0.624	0.824	0.004	0.979	0.998
Heschl L	0.451	0.002	0.003	0.350	0.021	0.072	0.275	0.075	0.942	-0.379	0.012	0.083	0.077	0.622	0.932
Heschl R	0.562	<0.001	<0.001	0.297	0.053	0.119	0.146	0.350	0.942	-0.292	0.057	0.194	0.090	0.567	0.932
Hippocampus L	0.492	0.001	0.001	0.492	0.001	0.011	-0.134	0.393	0.942	-0.299	0.051	0.181	0.069	0.660	0.937
Hippocampus R	0.483	0.001	0.002	0.376	0.013	0.055	-0.090	0.566	0.942	-0.219	0.159	0.367	0.021	0.896	0.998
Insula L	0.492	0.001	0.001	0.260	0.092	0.176	0.110	0.482	0.942	-0.255	0.099	0.267	0.086	0.582	0.932
Insula R	0.563	<0.001	<0.001	0.211	0.175	0.310	0.076	0.629	0.942	-0.201	0.197	0.422	-0.093	0.554	0.932
Lingual L	0.348	0.022	0.024	0.290	0.059	0.127	0.022	0.890	0.942	-0.189	0.225	0.439	0.118	0.449	0.932
Lingual R	0.327	0.032	0.034	0.303	0.049	0.119	-0.018	0.909	0.942	-0.197	0.205	0.430	0.001	0.993	0.998
Occipital Mid L	0.595	<0.001	<0.001	0.368	0.015	0.058	0.052	0.742	0.942	-0.338	0.026	0.123	0.233	0.133	0.681
Occipital Mid R	0.620	<0.001	<0.001	0.399	0.008	0.039	-0.070	0.656	0.942	-0.398	0.008	0.069	0.299	0.052	0.439
Occipital Sup L	0.483	0.001	0.002	0.306	0.046	0.119	-0.135	0.390	0.942	-0.250	0.106	0.267	0.272	0.077	0.441
Occipital Sup R	0.660	<0.001	<0.001	0.420	0.005	0.030	-0.184	0.237	0.942	-0.352	0.021	0.114	0.279	0.070	0.439

Olfactory L	0.451	0.002	0.003	0.373	0.014	0.055	0.106	0.501	0.942	-0.277	0.072	0.226	0.000	0.998	0.998
Olfactory R	0.443	0.003	0.004	0.394	0.009	0.040	0.151	0.335	0.942	-0.328	0.032	0.134	-0.085	0.587	0.932
Pallidum L	0.256	0.098	0.099	0.300	0.051	0.119	0.071	0.653	0.942	-0.309	0.044	0.175	0.096	0.538	0.932
Pallidum R	0.306	0.046	0.048	0.330	0.030	0.089	-0.033	0.835	0.942	-0.288	0.061	0.200	0.019	0.903	0.998
Paracentral Lobule L	0.522	<0.001	0.001	0.210	0.176	0.310	-0.150	0.337	0.942	-0.106	0.499	0.798	0.023	0.882	0.998
Paracentral Lobule R	0.468	0.002	0.002	0.077	0.623	0.772	-0.167	0.286	0.942	0.038	0.808	0.928	-0.003	0.984	0.998
ParaHippocampal L	0.505	0.001	0.001	0.004	0.980	0.988	-0.165	0.290	0.942	0.081	0.607	0.824	0.037	0.814	0.981
ParaHippocampal R	0.538	<0.001	0.001	0.053	0.733	0.830	-0.112	0.474	0.942	0.028	0.860	0.966	-0.042	0.789	0.978
Parietal Inf L	0.487	0.001	0.001	0.362	0.017	0.063	-0.041	0.796	0.942	-0.335	0.028	0.123	0.270	0.080	0.441
Parietal Inf R	0.579	<0.001	<0.001	0.464	0.002	0.017	-0.124	0.429	0.942	-0.396	0.009	0.069	0.377	0.013	0.439
Parietal Sup L	0.470	0.001	0.002	0.221	0.155	0.285	-0.117	0.454	0.942	-0.149	0.340	0.637	0.176	0.258	0.752
Parietal Sup R	0.534	<0.001	0.001	0.286	0.063	0.132	-0.121	0.438	0.942	-0.192	0.218	0.439	0.215	0.166	0.694
Postcentral L	0.580	<0.001	<0.001	0.411	0.006	0.032	-0.169	0.278	0.942	-0.337	0.027	0.123	0.319	0.037	0.439
Postcentral R	0.562	<0.001	<0.001	0.433	0.004	0.025	-0.211	0.174	0.942	-0.368	0.015	0.095	0.356	0.019	0.439
Precentral L	0.525	<0.001	0.001	0.274	0.075	0.147	-0.107	0.493	0.942	-0.190	0.223	0.439	0.227	0.143	0.681
Precentral R	0.557	<0.001	<0.001	0.340	0.026	0.081	-0.166	0.289	0.942	-0.266	0.084	0.247	0.296	0.054	0.439
Precuneus L	0.537	<0.001	0.001	0.181	0.245	0.399	-0.127	0.418	0.942	-0.074	0.636	0.824	0.070	0.655	0.937
Precuneus R	0.414	0.006	0.006	0.194	0.212	0.359	-0.080	0.610	0.942	-0.095	0.545	0.813	0.076	0.630	0.932
Putamen L	0.436	0.003	0.004	0.308	0.045	0.119	0.097	0.537	0.942	-0.214	0.167	0.378	0.064	0.682	0.952
Putamen R	0.506	0.001	0.001	0.283	0.066	0.135	0.140	0.370	0.942	-0.266	0.084	0.247	-0.028	0.860	0.998
Rectus L	0.508	0.001	0.001	-0.171	0.273	0.429	0.124	0.427	0.942	-0.011	0.943	0.975	0.148	0.345	0.820
Rectus R	0.519	<0.001	0.001	-0.137	0.381	0.540	0.199	0.200	0.942	-0.082	0.601	0.824	0.091	0.564	0.932
Rolandic Oper L	0.494	0.001	0.001	0.359	0.018	0.063	0.193	0.214	0.942	-0.389	0.010	0.072	0.196	0.207	0.729
Rolandic Oper R	0.498	0.001	0.001	0.324	0.034	0.096	0.170	0.276	0.942	-0.304	0.047	0.175	0.151	0.335	0.820
Supp Motor Area L	0.552	<0.001	<0.001	0.161	0.302	0.459	-0.212	0.172	0.942	-0.051	0.746	0.905	0.018	0.911	0.998
Supp Motor Area R	0.546	<0.001	0.001	0.111	0.479	0.613	-0.180	0.249	0.942	-0.011	0.945	0.975	-0.012	0.940	0.998
SupraMarginal L	0.478	0.001	0.002	0.488	0.001	0.011	0.100	0.525	0.942	-0.442	0.003	0.043	0.289	0.061	0.439
SupraMarginal R	0.489	0.001	0.001	0.604	<0.001	0.002	-0.064	0.684	0.942	-0.528	<0.001	0.024	0.346	0.023	0.439
Temporal Inf L	0.495	0.001	0.001	0.203	0.193	0.332	-0.057	0.717	0.942	-0.002	0.992	0.992	-0.155	0.322	0.820
Temporal Inf R	0.614	<0.001	<0.001	0.162	0.298	0.459	-0.071	0.649	0.942	0.013	0.933	0.975	0.101	0.520	0.932
Temporal Mid L	0.536	<0.001	0.001	0.313	0.041	0.112	0.132	0.397	0.942	-0.206	0.185	0.408	0.206	0.185	0.723
Temporal Mid R	0.624	<0.001	<0.001	0.416	0.006	0.030	0.054	0.733	0.942	-0.304	0.048	0.175	0.174	0.265	0.752
Temporal Pole Mid L	0.565	<0.001	<0.001	-0.148	0.345	0.497	0.323	0.035	0.942	0.024	0.878	0.966	0.049	0.756	0.978
Temporal Pole Mid R	0.594	<0.001	<0.001	-0.111	0.481	0.613	0.173	0.268	0.942	0.114	0.468	0.798	-0.074	0.636	0.932
Temporal Pole Sup L	0.536	<0.001	0.001	-0.053	0.735	0.830	0.008	0.958	0.969	0.111	0.477	0.798	0.126	0.419	0.932
Temporal Pole Sup R	0.579	<0.001	<0.001	0.057	0.716	0.830	0.174	0.264	0.942	-0.038	0.807	0.928	-0.009	0.956	0.998
Temporal Sup L	0.506	0.001	0.001	0.282	0.067	0.135	0.081	0.605	0.942	-0.237	0.125	0.306	0.097	0.536	0.932
Temporal Sup R	0.584	<0.001	<0.001	0.440	0.003	0.025	0.034	0.829	0.942	-0.362	0.017	0.100	0.200	0.198	0.727
Thalamus L	0.451	0.002	0.003	0.549	<0.001	0.006	0.027	0.864	0.942	-0.462	0.002	0.038	0.075	0.632	0.932
Thalamus R	0.333	0.029	0.031	0.502	0.001	0.011	-0.052	0.743	0.942	-0.416	0.006	0.055	0.043	0.787	0.978

The table presents AAL90 brain regions showing significant group differences in IC2, derived from mCCA + jICA, between mTBI patients and healthy controls.

Table S2. AAL90 complete correlations, p-values, and FDR for IC15.

	IC15														
	CBF			Fp			Ff			Fs			Ds		
ROI	ρ	p	FDR	ρ	p	FDR	ρ	p	FDR	ρ	p	FDR	ρ	p	FDR
Amygdala L	0.073	0.642	0.883	0.162	0.301	0.996	0.186	0.232	0.858	-0.248	0.109	0.990	0.199	0.201	0.457
Amygdala R	0.080	0.610	0.883	-0.012	0.941	0.996	0.296	0.054	0.853	-0.266	0.085	0.990	0.189	0.224	0.469
Angular L	0.096	0.540	0.883	0.079	0.614	0.996	0.187	0.229	0.858	-0.111	0.480	0.990	0.243	0.116	0.342
Angular R	0.092	0.558	0.883	0.017	0.913	0.996	0.055	0.726	0.948	-0.003	0.985	0.990	0.159	0.308	0.542
Calcarine L	0.208	0.181	0.883	-0.083	0.596	0.996	0.185	0.234	0.858	-0.048	0.760	0.990	0.371	0.014	0.215
Calcarine R	0.019	0.904	0.967	-0.119	0.447	0.996	0.164	0.294	0.858	-0.022	0.887	0.990	0.341	0.025	0.215
Caudate L	0.125	0.424	0.883	0.049	0.757	0.996	0.052	0.739	0.948	-0.075	0.635	0.990	0.396	0.009	0.215

Caudate R	0.029	0.852	0.967	0.140	0.372	0.996	-0.104	0.506	0.872	-0.102	0.516	0.990	0.346	0.023	0.215
Cingulum Ant L	0.094	0.548	0.883	0.006	0.971	0.996	0.126	0.421	0.858	-0.068	0.664	0.990	0.127	0.417	0.633
Cingulum Ant R	0.106	0.498	0.883	0.043	0.785	0.996	0.182	0.243	0.858	-0.137	0.380	0.990	0.077	0.623	0.772
Cingulum Mid L	0.146	0.349	0.883	0.099	0.526	0.996	0.219	0.158	0.858	-0.178	0.254	0.990	0.330	0.031	0.215
Cingulum Mid R	0.147	0.348	0.883	0.164	0.294	0.996	0.172	0.270	0.858	-0.207	0.183	0.990	0.253	0.101	0.331
Cingulum Post L	0.289	0.060	0.883	0.035	0.825	0.996	0.250	0.105	0.853	-0.161	0.302	0.990	0.299	0.052	0.228
Cingulum Post R	0.151	0.334	0.883	0.083	0.597	0.996	0.189	0.224	0.858	-0.183	0.240	0.990	0.334	0.029	0.215
Cuneus L	0.214	0.169	0.883	-0.035	0.826	0.996	0.189	0.224	0.858	-0.091	0.560	0.990	0.141	0.367	0.607
Cuneus R	0.019	0.902	0.967	-0.001	0.996	0.996	0.117	0.453	0.858	-0.058	0.710	0.990	0.173	0.268	0.523
Frontal Inf Oper L	0.163	0.296	0.883	0.054	0.731	0.996	0.318	0.038	0.853	-0.203	0.192	0.990	0.095	0.546	0.759
Frontal Inf Oper R	0.066	0.673	0.895	0.027	0.865	0.996	0.049	0.754	0.948	-0.070	0.658	0.990	0.131	0.404	0.623
Frontal Inf Orb L	0.126	0.421	0.883	-0.090	0.567	0.996	0.132	0.400	0.858	-0.021	0.896	0.990	0.050	0.751	0.843
Frontal Inf Orb R	0.109	0.488	0.883	-0.072	0.648	0.996	-0.032	0.839	0.972	-0.006	0.969	0.990	-0.030	0.849	0.883
Frontal Inf Tri L	0.109	0.486	0.883	0.088	0.576	0.996	0.244	0.115	0.853	-0.227	0.143	0.990	0.092	0.559	0.759
Frontal Inf Tri R	0.114	0.467	0.883	-0.039	0.803	0.996	0.054	0.732	0.948	-0.030	0.850	0.990	0.029	0.854	0.883
Frontal Med Orb L	0.093	0.552	0.883	-0.166	0.288	0.996	0.124	0.429	0.858	0.066	0.673	0.990	0.016	0.917	0.925
Frontal Med Orb R	0.099	0.529	0.883	-0.097	0.534	0.996	0.132	0.400	0.858	0.009	0.955	0.990	0.056	0.721	0.843
Frontal Mid L	0.100	0.525	0.883	0.123	0.433	0.996	-0.044	0.778	0.948	-0.118	0.452	0.990	0.027	0.863	0.883
Frontal Mid Orb L	0.106	0.497	0.883	-0.039	0.803	0.996	0.150	0.336	0.858	0.011	0.946	0.990	0.035	0.825	0.883
Frontal Mid Orb R	0.101	0.519	0.883	-0.074	0.638	0.996	0.104	0.505	0.872	0.002	0.990	0.990	-0.050	0.749	0.843
Frontal Mid R	0.055	0.726	0.920	0.050	0.748	0.996	0.009	0.953	0.990	-0.055	0.727	0.990	0.072	0.647	0.790
Frontal Sup L	0.176	0.259	0.883	0.103	0.511	0.996	-0.042	0.789	0.948	-0.093	0.553	0.990	-0.048	0.762	0.843
Frontal Sup Medial L	0.086	0.582	0.883	0.035	0.825	0.996	0.027	0.864	0.987	-0.046	0.769	0.990	0.028	0.860	0.883
Frontal Sup Medial R	0.086	0.585	0.883	0.117	0.454	0.996	-0.093	0.554	0.889	-0.079	0.614	0.990	-0.037	0.812	0.882
Frontal Sup Orb L	0.075	0.633	0.883	-0.087	0.580	0.996	0.184	0.237	0.858	-0.056	0.724	0.990	0.190	0.221	0.469
Frontal Sup Orb R	0.078	0.617	0.883	-0.063	0.688	0.996	-0.045	0.774	0.948	0.056	0.723	0.990	0.204	0.190	0.452
Frontal Sup R	0.105	0.504	0.883	0.069	0.660	0.996	-0.075	0.632	0.948	-0.045	0.777	0.990	0.047	0.767	0.843
Fusiform L	0.260	0.092	0.883	0.012	0.937	0.996	0.137	0.379	0.858	-0.110	0.484	0.990	0.159	0.308	0.542
Fusiform R	0.207	0.183	0.883	0.134	0.391	0.996	0.117	0.457	0.858	-0.171	0.274	0.990	0.254	0.101	0.331
Heschl L	0.021	0.896	0.967	-0.039	0.805	0.996	0.109	0.486	0.872	-0.016	0.920	0.990	0.155	0.321	0.554
Heschl R	0.047	0.767	0.935	-0.092	0.558	0.996	0.239	0.123	0.853	-0.031	0.846	0.990	0.060	0.702	0.835
Hippocampus L	0.265	0.086	0.883	-0.051	0.745	0.996	0.068	0.666	0.948	-0.004	0.981	0.990	0.184	0.238	0.486
Hippocampus R	0.183	0.239	0.883	0.037	0.815	0.996	-0.007	0.967	0.990	-0.023	0.885	0.990	0.245	0.113	0.342
Insula L	0.064	0.682	0.895	-0.027	0.863	0.996	0.285	0.064	0.853	-0.087	0.581	0.990	0.305	0.046	0.215
Insula R	0.029	0.854	0.967	-0.083	0.595	0.996	0.130	0.405	0.858	0.014	0.930	0.990	0.198	0.203	0.457
Lingual L	0.303	0.048	0.883	0.025	0.873	0.996	0.264	0.087	0.853	-0.177	0.257	0.990	0.257	0.096	0.331
Lingual R	0.268	0.082	0.883	0.012	0.937	0.996	0.314	0.040	0.853	-0.187	0.230	0.990	0.310	0.043	0.215
Occipital Mid L	0.123	0.430	0.883	0.006	0.971	0.996	0.143	0.359	0.858	-0.022	0.890	0.990	0.310	0.043	0.215
Occipital Mid R	0.148	0.343	0.883	-0.010	0.947	0.996	0.223	0.151	0.858	-0.037	0.816	0.990	0.065	0.678	0.817
Occipital Sup L	0.178	0.254	0.883	0.066	0.672	0.996	0.121	0.441	0.858	-0.124	0.427	0.990	0.135	0.389	0.612
Occipital Sup R	0.127	0.418	0.883	-0.064	0.683	0.996	0.160	0.307	0.858	-0.005	0.973	0.990	0.086	0.585	0.759
Olfactory L	0.079	0.613	0.883	0.117	0.453	0.996	0.130	0.405	0.858	-0.148	0.343	0.990	0.311	0.042	0.215
Olfactory R	0.003	0.986	0.997	0.174	0.264	0.996	0.045	0.774	0.948	-0.132	0.398	0.990	0.317	0.038	0.215
Pallidum L	0.213	0.171	0.883	-0.001	0.993	0.996	0.017	0.912	0.990	-0.020	0.900	0.990	0.360	0.018	0.215
Pallidum R	0.269	0.081	0.883	0.248	0.109	0.996	-0.320	0.036	0.853	0.126	0.420	0.990	0.322	0.035	0.215
Paracentral Lobule L	0.201	0.196	0.883	0.185	0.234	0.996	0.005	0.977	0.990	-0.184	0.238	0.990	0.277	0.072	0.277
Paracentral Lobule R	0.062	0.692	0.896	0.306	0.046	0.996	-0.130	0.407	0.858	-0.183	0.241	0.990	0.217	0.163	0.422
ParaHippocampal L	0.243	0.116	0.883	0.183	0.241	0.996	0.149	0.342	0.858	-0.195	0.210	0.990	0.138	0.378	0.607
ParaHippocampal R	0.187	0.230	0.883	0.196	0.209	0.996	0.251	0.105	0.853	-0.268	0.082	0.990	0.085	0.587	0.759
Parietal Inf L	0.052	0.739	0.920	0.236	0.128	0.996	-0.002	0.990	0.990	-0.170	0.276	0.990	0.236	0.128	0.363
Parietal Inf R	0.077	0.623	0.883	0.112	0.475	0.996	0.075	0.633	0.948	-0.090	0.565	0.990	0.084	0.592	0.759
Parietal Sup L	0.006	0.967	0.990	0.182	0.242	0.996	-0.092	0.555	0.889	-0.113	0.471	0.990	0.122	0.436	0.651
Parietal Sup R	-0.021	0.896	0.967	0.091	0.561	0.996	0.009	0.952	0.990	-0.084	0.593	0.990	0.052	0.743	0.843
Postcentral L	0.067	0.671	0.895	0.179	0.252	0.996	-0.020	0.901	0.990	-0.138	0.377	0.990	0.168	0.282	0.531
Postcentral R	0.085	0.589	0.883	0.069	0.662	0.996	-0.054	0.730	0.948	-0.038	0.811	0.990	0.114	0.465	0.682

Precentral L	0.126	0.423	0.883	0.151	0.334	0.996	-0.040	0.799	0.948	-0.137	0.382	0.990	0.167	0.283	0.531
Precentral R	0.136	0.383	0.883	0.056	0.722	0.996	-0.009	0.954	0.990	-0.045	0.773	0.990	0.086	0.583	0.759
Precuneus L	0.161	0.301	0.883	0.163	0.295	0.996	0.010	0.947	0.990	-0.164	0.294	0.990	0.293	0.057	0.237
Precuneus R	0.038	0.810	0.964	0.151	0.334	0.996	0.022	0.887	0.990	-0.138	0.379	0.990	0.176	0.258	0.516
Putamen L	0.151	0.333	0.883	-0.122	0.436	0.996	0.058	0.712	0.948	-0.013	0.932	0.990	0.378	0.012	0.215
Putamen R	0.106	0.497	0.883	0.013	0.935	0.996	-0.114	0.468	0.858	0.108	0.491	0.990	0.351	0.021	0.215
Rectus L	0.084	0.593	0.883	0.024	0.878	0.996	0.126	0.422	0.858	-0.114	0.467	0.990	0.373	0.014	0.215
Rectus R	0.076	0.627	0.883	0.108	0.492	0.996	0.093	0.554	0.889	-0.156	0.319	0.990	0.384	0.011	0.215
Rolandic Oper L	0.013	0.934	0.967	0.017	0.912	0.996	0.294	0.056	0.853	-0.168	0.282	0.990	0.192	0.217	0.469
Rolandic Oper R	-0.027	0.863	0.967	-0.018	0.909	0.996	0.237	0.126	0.853	-0.126	0.419	0.990	0.207	0.183	0.446
Supp Motor Area L	0.110	0.483	0.883	0.223	0.150	0.996	-0.038	0.808	0.948	-0.197	0.205	0.990	0.336	0.028	0.215
Supp Motor Area R	0.114	0.466	0.883	0.317	0.039	0.996	-0.166	0.289	0.858	-0.213	0.170	0.990	0.247	0.110	0.342
SupraMarginal L	0.121	0.441	0.883	-0.033	0.834	0.996	0.246	0.112	0.853	-0.064	0.683	0.990	0.091	0.563	0.759
SupraMarginal R	0.111	0.480	0.883	-0.045	0.776	0.996	0.004	0.979	0.990	0.059	0.705	0.990	0.161	0.303	0.542
Temporal Inf L	0.199	0.202	0.883	-0.004	0.980	0.996	0.135	0.389	0.858	-0.141	0.367	0.990	0.269	0.082	0.300
Temporal Inf R	0.163	0.296	0.883	-0.039	0.803	0.996	0.170	0.277	0.858	-0.143	0.361	0.990	0.212	0.173	0.435
Temporal Mid L	0.079	0.615	0.883	-0.029	0.853	0.996	0.193	0.214	0.858	-0.061	0.700	0.990	0.232	0.134	0.369
Temporal Mid R	0.112	0.475	0.883	-0.017	0.913	0.996	0.051	0.744	0.948	0.008	0.958	0.990	0.140	0.371	0.607
Temporal Pole Mid L	0.016	0.919	0.967	0.352	0.021	0.996	0.065	0.677	0.948	-0.394	0.009	0.784	-0.015	0.925	0.925
Temporal Pole Mid R	0.052	0.742	0.920	0.163	0.296	0.996	0.092	0.556	0.889	-0.253	0.102	0.990	-0.081	0.608	0.764
Temporal Pole Sup L	0.000	0.999	0.999	0.150	0.337	0.996	0.177	0.256	0.858	-0.235	0.130	0.990	0.083	0.595	0.759
Temporal Pole Sup R	-0.014	0.927	0.967	0.129	0.409	0.996	0.115	0.462	0.858	-0.230	0.137	0.990	-0.090	0.564	0.759
Temporal Sup L	0.035	0.826	0.967	-0.010	0.947	0.996	0.217	0.162	0.858	-0.091	0.561	0.990	0.226	0.144	0.384
Temporal Sup R	0.045	0.775	0.935	-0.021	0.892	0.996	-0.073	0.643	0.948	0.030	0.849	0.990	0.137	0.379	0.607
Thalamus L	0.474	0.001	0.115	-0.035	0.822	0.996	0.062	0.692	0.948	-0.010	0.951	0.990	0.306	0.046	0.215
Thalamus R	0.288	0.061	0.883	0.043	0.785	0.996	0.089	0.572	0.899	-0.095	0.543	0.990	0.283	0.066	0.264

The table presents AAL90 brain regions showing significant group differences in IC15, derived from mCCA + jICA, between mTBI patients and healthy controls.

Table S3. Complete correlations between GOS-E and IVIM/ASL metrics inside the ROIs of the AAL90 atlas. Table includes ϱ, p-values, and FDR values.

	CBF			Fp			Ff			Fs			Ds		
**ROI**	ϱ	**p**	**FDR**	ϱ	**p**	**FDR**	ϱ	**p**	**FDR**	ϱ	**p**	**FDR**	ϱ	**p**	**FDR**
Amygdala L	0.087	0.723	0.969	0.224	0.356	0.857	-0.194	0.425	0.923	-0.008	0.975	0.975	0.499	0.030	0.169
Amygdala R	0.136	0.577	0.969	-0.034	0.889	0.961	-0.133	0.587	0.939	0.146	0.552	0.866	0.436	0.062	0.227
Angular L	0.102	0.678	0.969	0.249	0.304	0.857	0.174	0.475	0.923	-0.285	0.236	0.629	0.267	0.270	0.416
Angular R	0.154	0.530	0.969	0.130	0.595	0.961	0.297	0.217	0.923	-0.300	0.211	0.629	0.362	0.128	0.278
Calcarine L	-0.275	0.255	0.969	0.218	0.369	0.857	0.321	0.181	0.923	-0.360	0.130	0.629	0.382	0.107	0.272
Calcarine R	-0.050	0.838	0.969	0.390	0.099	0.702	0.501	0.029	0.850	-0.539	0.017	0.305	0.249	0.304	0.445
Caudate L	0.136	0.578	0.969	-0.030	0.903	0.961	-0.075	0.760	0.972	0.084	0.734	0.897	0.301	0.210	0.342
Caudate R	0.160	0.512	0.969	0.346	0.147	0.702	-0.358	0.133	0.923	-0.133	0.589	0.866	0.364	0.125	0.278
Cingulum Ant L	0.037	0.880	0.969	-0.022	0.928	0.961	0.088	0.719	0.972	-0.028	0.909	0.962	0.340	0.154	0.294
Cingulum Ant R	0.012	0.961	0.972	0.115	0.640	0.961	0.058	0.814	0.972	-0.129	0.600	0.866	0.409	0.082	0.249
Cingulum Mid L	-0.024	0.924	0.969	0.025	0.920	0.961	0.166	0.498	0.923	-0.087	0.722	0.897	0.491	0.033	0.170
Cingulum Mid R	0.105	0.669	0.969	0.138	0.573	0.961	0.127	0.604	0.949	-0.158	0.519	0.866	0.587	0.008	0.109
Cingulum Post L	-0.214	0.380	0.969	0.224	0.357	0.857	0.201	0.409	0.923	-0.295	0.221	0.629	0.297	0.216	0.346
Cingulum Post R	-0.004	0.988	0.988	0.276	0.253	0.798	0.152	0.535	0.923	-0.304	0.206	0.629	0.196	0.421	0.553
Cuneus L	-0.256	0.289	0.969	0.275	0.254	0.798	0.183	0.453	0.923	-0.285	0.236	0.629	0.320	0.182	0.313
Cuneus R	-0.038	0.879	0.969	0.435	0.063	0.702	0.441	0.058	0.850	-0.498	0.030	0.412	0.381	0.108	0.272
Frontal Inf Oper L	0.136	0.579	0.969	0.328	0.171	0.702	-0.019	0.939	0.972	-0.227	0.351	0.772	0.662	0.002	0.109
Frontal Inf Oper R	0.162	0.508	0.969	0.093	0.704	0.961	0.415	0.078	0.850	-0.221	0.362	0.778	0.371	0.118	0.274
Frontal Inf Orb L	0.127	0.603	0.969	0.048	0.844	0.961	0.261	0.280	0.923	-0.159	0.516	0.866	0.358	0.133	0.278
Frontal Inf Orb R	0.168	0.491	0.969	0.150	0.539	0.952	0.154	0.528	0.923	-0.307	0.202	0.629	-0.061	0.804	0.869
Frontal Inf Tri L	0.043	0.862	0.969	0.214	0.380	0.857	0.162	0.507	0.923	-0.164	0.501	0.866	0.477	0.039	0.175

Frontal Inf Tri R	0.074	0.762	0.969	-0.060	0.807	0.961	0.558	0.013	0.850	-0.078	0.751	0.905	0.208	0.392	0.536
Frontal Med Orb L	0.176	0.471	0.969	-0.149	0.543	0.952	-0.024	0.923	0.972	0.118	0.631	0.895	0.233	0.336	0.485
Frontal Med Orb R	-0.018	0.943	0.969	-0.044	0.859	0.961	0.019	0.937	0.972	0.019	0.938	0.971	0.261	0.281	0.426
Frontal Mid L	0.116	0.636	0.969	0.197	0.420	0.880	-0.206	0.397	0.923	-0.104	0.671	0.897	0.386	0.102	0.272
Frontal Mid Orb L	0.058	0.814	0.969	0.099	0.686	0.961	0.049	0.841	0.972	-0.031	0.900	0.962	0.212	0.384	0.536
Frontal Mid Orb R	0.087	0.723	0.969	-0.095	0.699	0.961	0.220	0.365	0.923	-0.036	0.885	0.962	0.092	0.707	0.778
Frontal Mid R	0.118	0.629	0.969	-0.093	0.704	0.961	0.281	0.244	0.923	0.025	0.919	0.962	0.146	0.550	0.682
Frontal Sup L	0.141	0.564	0.969	0.071	0.773	0.961	-0.096	0.697	0.972	-0.034	0.891	0.962	0.325	0.175	0.313
Frontal Sup Medial L	0.205	0.399	0.969	-0.058	0.812	0.961	-0.095	0.699	0.972	0.087	0.725	0.897	0.206	0.399	0.536
Frontal Sup Medial R	0.147	0.549	0.969	-0.108	0.661	0.961	-0.079	0.747	0.972	0.128	0.601	0.866	0.279	0.248	0.389
Frontal Sup Orb L	0.076	0.757	0.969	0.045	0.855	0.961	0.037	0.880	0.972	-0.049	0.844	0.962	0.121	0.621	0.716
Frontal Sup Orb R	0.076	0.756	0.969	0.089	0.718	0.961	0.007	0.977	0.988	-0.096	0.697	0.897	0.092	0.708	0.778
Frontal Sup R	0.076	0.757	0.969	-0.100	0.683	0.961	0.229	0.346	0.923	0.033	0.894	0.962	0.027	0.912	0.925
Fusiform L	-0.199	0.413	0.969	0.119	0.628	0.961	0.032	0.895	0.972	-0.104	0.672	0.897	0.371	0.118	0.274
Fusiform R	0.097	0.692	0.969	0.051	0.836	0.961	0.136	0.577	0.939	-0.145	0.554	0.866	0.441	0.059	0.225
Heschl L	0.038	0.877	0.969	0.325	0.175	0.702	0.144	0.557	0.932	-0.333	0.164	0.629	0.168	0.491	0.618
Heschl R	0.032	0.896	0.969	0.621	0.005	0.404	0.307	0.201	0.923	-0.641	0.003	0.095	-0.017	0.944	0.944
Hippocampus L	-0.084	0.731	0.969	0.221	0.364	0.857	-0.036	0.883	0.972	-0.101	0.681	0.897	0.596	0.007	0.109
Hippocampus R	0.180	0.460	0.969	-0.018	0.941	0.963	0.060	0.807	0.972	-0.033	0.892	0.962	0.552	0.014	0.109
Insula L	0.175	0.475	0.969	0.254	0.293	0.857	0.107	0.663	0.972	-0.262	0.278	0.692	0.569	0.011	0.109
Insula R	0.148	0.547	0.969	0.451	0.052	0.702	0.167	0.496	0.923	-0.457	0.049	0.412	0.204	0.402	0.536
Lingual L	-0.274	0.257	0.969	0.013	0.958	0.969	0.194	0.426	0.923	-0.143	0.560	0.866	0.467	0.044	0.175
Lingual R	-0.020	0.936	0.969	0.095	0.700	0.961	0.421	0.073	0.850	-0.291	0.226	0.629	0.428	0.067	0.237
Occipital Mid L	0.039	0.875	0.969	0.343	0.151	0.702	0.313	0.191	0.923	-0.443	0.058	0.412	0.130	0.597	0.709
Occipital Mid R	-0.016	0.947	0.969	0.180	0.462	0.924	0.181	0.460	0.923	-0.325	0.174	0.629	0.182	0.455	0.588
Occipital Sup L	-0.018	0.941	0.969	0.375	0.114	0.702	0.160	0.514	0.923	-0.343	0.151	0.629	0.344	0.149	0.292
Occipital Sup R	0.172	0.482	0.969	0.347	0.146	0.702	0.242	0.319	0.923	-0.368	0.121	0.629	0.467	0.044	0.175
Olfactory L	-0.066	0.790	0.969	0.351	0.140	0.702	0.058	0.815	0.972	-0.216	0.374	0.785	0.135	0.581	0.709
Olfactory R	-0.089	0.717	0.969	0.433	0.064	0.702	0.049	0.842	0.972	-0.255	0.292	0.695	0.056	0.820	0.869
Pallidum L	0.083	0.735	0.969	0.075	0.761	0.961	0.091	0.712	0.972	-0.159	0.516	0.866	0.588	0.008	0.109
Pallidum R	0.130	0.597	0.969	-0.208	0.392	0.862	0.043	0.860	0.972	0.166	0.498	0.866	0.573	0.010	0.109
Paracentral Lobule L	0.160	0.513	0.969	-0.004	0.986	0.986	0.250	0.301	0.923	-0.158	0.517	0.866	0.172	0.482	0.615
Paracentral Lobule R	0.315	0.189	0.969	0.320	0.182	0.702	0.163	0.506	0.923	-0.324	0.175	0.629	0.470	0.043	0.175
ParaHippocampal L	0.048	0.845	0.969	-0.043	0.863	0.961	0.072	0.770	0.972	-0.033	0.894	0.962	0.535	0.018	0.124
ParaHippocampal R	0.186	0.447	0.969	0.069	0.780	0.961	0.241	0.321	0.923	-0.178	0.465	0.866	0.322	0.179	0.313
Parietal Inf L	0.155	0.527	0.969	0.301	0.210	0.734	0.034	0.890	0.972	-0.260	0.283	0.692	0.384	0.105	0.272
Parietal Inf R	0.130	0.596	0.969	0.164	0.502	0.941	0.242	0.317	0.923	-0.281	0.243	0.629	0.575	0.010	0.109
Parietal Sup L	0.106	0.666	0.969	0.368	0.121	0.702	0.037	0.881	0.972	-0.304	0.205	0.629	0.350	0.142	0.284
Parietal Sup R	0.141	0.565	0.969	0.146	0.552	0.952	0.186	0.445	0.923	-0.200	0.411	0.822	0.470	0.042	0.175
Postcentral L	0.064	0.795	0.969	0.215	0.376	0.857	-0.041	0.868	0.972	-0.175	0.475	0.866	0.254	0.294	0.439
Postcentral R	0.079	0.749	0.969	0.159	0.514	0.943	0.173	0.478	0.923	-0.243	0.316	0.731	0.381	0.108	0.272
Precentral L	0.021	0.933	0.969	0.164	0.503	0.941	-0.157	0.521	0.923	-0.091	0.711	0.897	0.320	0.181	0.313
Precentral R	0.036	0.884	0.969	0.029	0.907	0.961	0.178	0.467	0.923	-0.093	0.704	0.897	0.415	0.077	0.249
Precuneus L	-0.113	0.646	0.969	0.415	0.077	0.702	0.171	0.483	0.923	-0.361	0.129	0.629	0.383	0.105	0.272
Precuneus R	0.037	0.881	0.969	0.396	0.094	0.702	0.296	0.219	0.923	-0.405	0.085	0.537	0.376	0.113	0.274
Putamen L	0.085	0.729	0.969	0.179	0.464	0.924	-0.382	0.106	0.850	0.333	0.163	0.629	0.496	0.031	0.169
Putamen R	0.129	0.598	0.969	-0.050	0.839	0.961	-0.389	0.100	0.850	0.438	0.061	0.412	0.549	0.015	0.109
Rectus L	0.046	0.852	0.969	-0.072	0.771	0.961	0.052	0.832	0.972	-0.012	0.962	0.975	-0.050	0.839	0.869
Rectus R	-0.049	0.841	0.969	0.060	0.807	0.961	-0.014	0.956	0.978	-0.026	0.917	0.962	0.027	0.914	0.925
Rolandic Oper L	0.117	0.633	0.969	0.333	0.163	0.702	0.055	0.823	0.972	-0.300	0.212	0.629	0.562	0.012	0.109
Rolandic Oper R	0.117	0.634	0.969	0.535	0.018	0.702	0.471	0.042	0.850	-0.603	0.006	0.138	0.416	0.076	0.249
Supp Motor Area L	0.067	0.787	0.969	-0.038	0.878	0.961	0.001	0.996	0.996	0.032	0.895	0.962	0.123	0.617	0.716
Supp Motor Area R	0.212	0.383	0.969	0.052	0.833	0.961	-0.105	0.670	0.972	0.010	0.967	0.975	0.228	0.348	0.494
SupraMarginal L	0.098	0.689	0.969	0.336	0.160	0.702	0.066	0.788	0.972	-0.343	0.151	0.629	0.306	0.203	0.337
SupraMarginal R	0.171	0.483	0.969	0.215	0.377	0.857	0.353	0.138	0.923	-0.453	0.052	0.412	0.511	0.025	0.159

Temporal Inf L	0.122	0.619	0.969	0.264	0.275	0.835	0.023	0.926	0.972	-0.211	0.386	0.791	0.306	0.202	0.337
Temporal Inf R	0.112	0.649	0.969	0.175	0.473	0.924	0.142	0.561	0.932	-0.235	0.332	0.749	-0.058	0.812	0.869
Temporal Mid L	0.120	0.625	0.969	0.406	0.084	0.702	0.247	0.309	0.923	-0.440	0.059	0.412	0.355	0.136	0.278
Temporal Mid R	0.158	0.517	0.969	0.495	0.031	0.702	0.458	0.049	0.850	-0.653	0.002	0.095	0.119	0.626	0.716
Temporal Pole Mid L	0.195	0.423	0.969	0.026	0.916	0.961	0.169	0.488	0.923	-0.145	0.554	0.866	0.358	0.133	0.278
Temporal Pole Mid R	0.323	0.178	0.969	0.310	0.196	0.719	0.250	0.301	0.923	-0.477	0.039	0.412	0.112	0.649	0.732
Temporal Pole Sup L	0.189	0.439	0.969	0.071	0.772	0.961	0.066	0.787	0.972	-0.132	0.590	0.866	0.409	0.082	0.249
Temporal Pole Sup R	0.226	0.352	0.969	0.297	0.217	0.734	0.189	0.438	0.923	-0.462	0.047	0.412	0.051	0.834	0.869
Temporal Sup L	0.164	0.501	0.969	0.319	0.184	0.702	0.176	0.471	0.923	-0.354	0.137	0.629	0.327	0.172	0.313
Temporal Sup R	0.173	0.478	0.969	0.488	0.034	0.702	0.330	0.168	0.923	-0.639	0.003	0.095	0.133	0.588	0.709
Thalamus L	-0.120	0.624	0.969	0.203	0.405	0.869	-0.399	0.091	0.850	0.130	0.597	0.866	0.587	0.008	0.109
Thalamus R	-0.017	0.945	0.969	0.241	0.321	0.857	-0.439	0.060	0.850	0.114	0.643	0.897	0.551	0.015	0.109

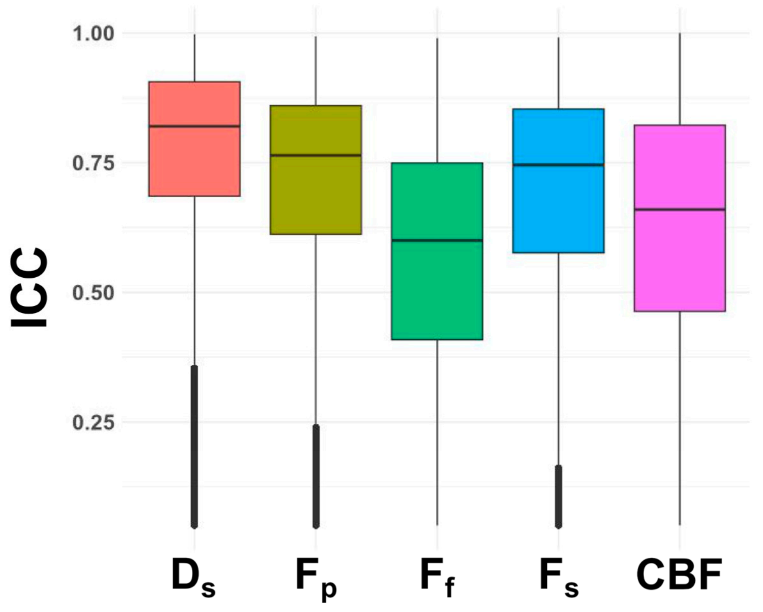

MAP	Mean (SD)
Ds	0.77 (0.19)
Fp	0.71 (0.20)
Ff	0.56 (0.24)
Fs	0.69 (0.21)
CBF	0.66 (0.26)

Figure S1. Test–retest reliability of IVIM-derived parameters and ASL-derived CBF in 11 healthy controls. Boxplots show the distribution of voxel-wise intraclass correlation coefficients (ICC) for each parameter, and the table summarizes mean ICC values (± Standard Deviation; SD).
